# A Novel Discovery of Microorganisms Associated with Certain Seaweed Species from the Cat Ba Archipelago Area, Vietnam: Isolation, Identification and Exploration of Their Antimicrobial Activities

**DOI:** 10.3390/microorganisms14091994

**Published:** 2026-09-09

**Authors:** Thanh Tien Tran, Nga Thi Nguyen, Phuong Thi Nguyen, Anh Hien Le, Le Na Thi Nguyen, Lien Thuy Nguyen, Duc Anh Nguyen, Phuong Ha Vu, Thao Kim Nu Nguyen, Tung Thanh Nguyen, Ngoc Anh Truong, Luyen Thuy Thi Bui, Hai The Pham

**Affiliations:** 1 Research Group for Physiology and Applications of Microorganisms (PHAM Group), GREENLAB, Center for Life Science Research (CELIFE), Faculty of Biology, VNU University of Science, Vietnam National University Hanoi, 334 Nguyen Trai, Hanoi 100000, Vietnam; trantienthanh_t65@hus.edu.vn (T.T.T.); nguyenthinga_t66@hus.edu.vn (N.T.N.); nguyenthiphuong2_t66@hus.edu.vn (P.T.N.);; 2 Department of Biochemistry and Molecular Biology, Faculty of Biology, VNU University of Science, Vietnam National University Hanoi, 334 Nguyen Trai, Hanoi 100000, Vietnam; 3 Department of Plant Science, Faculty of Biology, VNU University of Science, Vietnam National University Hanoi, 334 Nguyen Trai, Hanoi 100000, Vietnam; thuylienhus@gmail.com (L.T.N.);; 4 Department of Cell Biology, Faculty of Biology, VNU University of Science, Vietnam National University Hanoi, 334 Nguyen Trai, Hanoi 100000, Vietnam; 5 Faculty of Pharmacognosy and Traditional Medicine, Hanoi University of Pharmacy, 13-15 Le Thanh Tong, Hanoi 100000, Vietnamtruonganhngoc01082004@gmail.com (N.A.T.); 6 Faculty of Pharmaceutical Chemistry and Technology, Hanoi University of Pharmacy, Hanoi 100000, Vietnam; luyenbtt@hup.edu.vn; 7 Department of Microbiology, Faculty of Biology, VNU University of Science, Vietnam National University Hanoi, 334 Nguyen Trai, Hanoi 100000, Vietnam

**Keywords:** macroalgae, marine microorganisms, bioprospecting, *Bacillus velezensis*, LC–HRMS/MS, bioactive compounds

## Abstract

Marine macroalgae, or seaweed, are an attractive source of novel natural products, which are in many cases not only produced by the algae themselves but also by their associated microorganisms. Therefore, the search for novel products should not only be aimed at the algae but also their symbionts. In this study, we attempted to investigate culturable microorganisms associated with nine marine macroalgal hosts collected from the Cat Ba Archipelago area (Vietnam), which is known to have significant biodiversity value. More specifically, such microorganisms were isolated by using standard plating methods with eight media under varied nutrient and salinity conditions, screened for antimicrobial activity via agar diffusion assays and MIC/MBC determination methods; those with the most remarkable activities were identified based on their morphology, 16S rRNA gene sequences, and housekeeping gene *rpoB* sequences if necessary. We obtained a total of 331 isolates, among which 15 bacterial isolates exhibited detectable antimicrobial activity against at least one of the four test microorganisms. The isolates displaying notable antimicrobial activities were affiliated predominantly with *Bacillus* species, together with representatives of *Virgibacillus*, *Exiguobacterium*, *Alcaligenes* and *Serratia*. Interestingly, almost all of the associations of those bacteria with the seaweed hosts have not yet been reported. Among those isolates, *Bacillus velezensis* S.002, found to be associated with the red alga host *Gelidium crinale* for the first time, exhibited the most remarkable antibacterial activity, and the ethylacetate extract of its culture showed the lowest MIC values against both *B. subtilis* and *X. oryzae*. The extract was therefore fractionated and the fraction exhibiting pronounced antibacterial activities was analyzed by LC–HRMS to putatively annotate its metabolites. The results revealed that, in addition to canonical lipopeptides, S.002 could produce several other metabolites that have merely been reported for *Bacillus velezensis*, including some alkaloids and a unique bioactive amino acid, which might synergistically contribute to the antimicrobial efficacy of the strain. Collectively, the results highlight Cat Ba macroalgae as promising sources of bioactive microorganisms, which in turn can be a promising source of bioactive compounds, produced as part of the host–microbe interactions.

## 1. Introduction

Marine macroalgae, commonly referred to as seaweed, are among the most important habitat-forming primary producers in coastal ecosystems. As abundant and diverse sessile multicellular photosynthetic eukaryotes, they play central roles in ecosystem functioning by enhancing habitat complexity, supporting food webs, and contributing substantially to primary productivity in marine systems worldwide [1]. Seaweeds, including brown, red, and green algae, also synthesize a wide range of structural and bioactive compounds, such as proteins, lipids, carbohydrates, and other metabolites, which have attracted considerable interest for agricultural, cosmeceutical, pharmaceutical, and biotechnological applications [2]. In addition, although seaweeds have long been used as food, especially in Asia, their broader industrial use has expanded more recently in response to increasing interest in sustainable biomass resources, including applications related to biofuel production and food security [3].

The ecological performance and practical value of seaweeds are strongly influenced by their interactions with associated microbial communities. Seaweed-associated microbiota comprise diverse organisms inhabiting algal surfaces and tissues, including bacteria, archaea, fungi, protists, and viruses. These microorganisms can positively influence macroalgal morphogenesis, growth, development, and stress tolerance, while also contributing to host protection against harmful colonizers, although in some cases they can also exert detrimental effects, such as disease development [4]. Together, the host and its associated microbiota form a holobiont, an integrated ecological unit maintained through specialized symbiotic interactions that are important for the functioning of both [4]. Therefore, the ecological and biotechnological significance of seaweeds cannot be fully understood without considering the microorganisms associated with them [5].

Seaweed-associated microorganisms are also an important group of marine microorganisms which are increasingly regarded as a promising source of biologically active compounds owing to their capacity to produce structurally diverse secondary metabolites [6]. This capacity stems from the remarkable heterogeneity of marine habitats and the still limited understanding of the metabolic potential harbored by marine microbes. Marine bacteria have already attracted substantial biotechnological interest due to their ability to produce biosurfactants, cold-adapted enzymes, and structurally diverse secondary metabolites with potential applications in industry and medicine [7,8]. At the same time, the global rise of antimicrobial resistance has intensified the search for novel natural products capable of complementing or replacing conventional antibiotics [9]. In this context, marine microorganisms, including those associated with macroalgae, represent an attractive reservoir of antimicrobial metabolites and therefore a valuable resource for the discovery of novel antibiotic-producing strains [10]. Therefore, the search for novel marine microorganisms as well as novel host–microbe relationships would obviously increase the chance of discovering novel products.

Despite the growing recognition of seaweed-associated microorganisms as ecologically important and biotechnologically promising objects, culture-based studies on these microbial communities remain comparatively limited and their results showed an uneven distribution of various microbial groups across host taxa and geographic regions [11,12]. In Vietnam, there are only a few available studies reporting the isolation of marine algae-associated fungi with antimicrobial, cytotoxic, and hemolytic activities, or highlighting the diversity and enzymatic potential of seaweed-associated bacteria, for example from Nha Trang Bay [13,14]. However, these studies suggest an emerging rather than comprehensive research landscape, and information is still limited regarding host-dependent recovery patterns, the influence of isolation media, and the antimicrobial potential of cultured microorganisms associated with different macroalgal hosts. Thus, more systematic investigation is needed to understand profoundly seaweed-associated microorganisms in Vietnam and to identify those with antimicrobial potential values. Therefore, this study aims to isolate culturable microorganisms associated with nine marine macroalgal hosts collected from the Cat Ba Archipelago area, Hai Phong, Vietnam, screen them for antimicrobial activity, identify the most promising isolates, and preliminarily determine the composition and the structures of their extracellular bioactive metabolites. We selected algal samples from the Cat Ba Archipelago area as this area is known to have significant biodiversity value; furthermore, research on seaweed-associated microorganisms in this area is very limited [15]. We expected that different macroalgal hosts would harbor distinct microbes and that these underexplored marine microorganisms could represent a promising source of antimicrobial metabolites and potentially new antimicrobial leads.

## 2. Materials and Methods

### 2.1. Seaweed Samples

Nine different seaweed samples were used for the isolation of associating microorganisms in this study. They were collected from several coastal sites in the Cat Ba Archipelago area, Hai Phong, Vietnam (see Table S1 for detailed information), handpicked by researchers wearing sterile gloves. Once collected, each sample was rinsed 2–3 times with sea water from the same location and subsequently stored in dark sterile plastic bags filled with sea water, maintained at 4 °C under cool conditions during transport, and utilized for experiments within 48 h after collection. They were identified as *Ulva flexuosa* (Wulfen 1803), *Valonia fastigiata* (Harvey ex J. Agardh 1887), *Caulerpa racemosa* (Forsskål J. Agardh 1873), *Chaetomorpha linum* ((O. F. Müller) Kützing 1845), *Cladophora aokii* (Yamada 1925), *Gelidium crinale* ((Turner) J. V. Lamour 1825), *Gracilaria tenuistipitata* (Chang & B.M.Xia 1976), *Dermonema pulvinatum* ((Grunow) Fan 1962), and *Centroceras clavulatum* ((C. Argardh) Montagne 1846), as previously reported by Lien and Thao [16]. The first five are green algae while the remaining four are red algae.

### 2.2. Isolation of Seaweed-Associated Microorganisms

Seaweed samples were washed three times with sterile 0.9% (*w*/*v*) NaCl solution in order to remove free-living and loosely attached bacteria. Subsequently, the firmly attached epiphytic microorganisms were collected by vortexing 1 g of seaweed biomass in 9 mL sterile water for 5 min. For endophytic microorganisms, seaweed tissues were surface-sterilized with 96% ethanol for 3 min, 3% H_2_O_2_ for 3 min, and 1% NaClO for 3 min. The sterilized tissues were subsequently rinsed thoroughly with sterile distilled water, and the final rinse was plated to confirm the effectiveness of surface sterilization. The surface-sterilized tissues were then homogenized in sterile saline [17]. Microorganisms were isolated by serial dilution using sterile NaCl 0.9% solution and plating in triplicate on eight agar media under different nutrient and salinity conditions, including Luria–Bertani agar (LB), LB supplemented with 3% (*w*/*v*) NaCl (LB 3%), yeast–sucrose agar (YS), YS supplemented with 3% (*w*/*v*) NaCl (YS 3%), potato dextrose agar (PDA), PDA supplemented with 3% (*w*/*v*) NaCl (PDA 3%), Marine Agar, and starch casein agar (SCA). The plates were incubated at 30 °C for at least 7 days. Morphologically distinct colonies were selected and purified by repeated streaking. To standardize morphological comparison and minimize redundancy among isolates recovered from different isolation media, purified isolates suspected to be bacteria were subsequently cultured on LB agar containing 1% (*w*/*v*) NaCl, actinomycetes on YS agar, and fungi on PDA. Colony morphology was compared under these standardized group-specific conditions, and isolates showing indistinguishable colony characteristics were progressively excluded. Suspected overlapping isolates were also checked by later gene sequence analyses. The resulting isolates were preserved at −80 °C in 30% (*v*/*v*) glycerol.

### 2.3. Methods of Screening for Antimicrobial Activity

#### 2.3.1. Test Microorganisms and Their Growth Conditions

Four test microorganisms were used in this study, including two bacterial strains (*Bacillus subtilis* VS provided by the Department of Microbiology, Faculty of Biology, VNU University of Science, and *Xanthomonas oryzae* PAM100 in the collection of GREENLAB, Center for Life Science Research, Faculty of Biology, VNU University of Science), and two fungal strains (*Fusarium oxysporum* PAM50 and *Phomopsis asparagi* PAM52, both in the collection of GREENLAB). To prepare the cultures of the test bacteria, *X. oryzae* was grown in potato sucrose (PS) broth at 30 °C and 200 rpm for 24 h, whereas *B. subtilis* was grown in 1% Luria–Bertani (LB) broth under the same conditions for 12 h. As for the test fungal strains, they were cultivated in potato dextrose (PD) broth at 30 °C and 200 rpm for 72 h.

#### 2.3.2. Screening for Antimicrobial Activity

The isolates were cultivated under standardized microorganism group-specific conditions for antimicrobial screening. Bacterial isolates were cultured in LB broth containing 1% (*w*/*v*) NaCl, actinomycetes in YS broth, and fungal isolates in potato dextrose broth (PDB). All cultures were incubated at 30 °C with shaking at 200 rpm for 60 h prior to antimicrobial activity testing.

The isolates were screened for antimicrobial activity via the agar well diffusion method. Briefly, 100 µL of each test microorganism culture was spread onto the surface of the corresponding agar medium, i.e., PS agar for *X. oryzae*, 1% LB agar for *B. subtilis*, and PD agar for *F. oxysporum* and *P. asparagi*. After the agar surface had dried, 5 mm diameter wells were made in the plate using a sterile punch. The culture broth of each tested isolate was centrifuged at 6000 rpm for 10 min, and the clarified supernatant was collected and added into each well and allowed to diffuse at 4 °C for 1 h [18]. The plates were subsequently incubated at 30 °C for 24 h for the test bacteria and 72 h for the test fungi. The level of antimicrobial activity was determined by the diameter of the clear zone around each well, including the 5 mm well diameter. Erythromycin (10 mg/mL) was used as the positive control for the antibacterial assays, whereas salicylic acid (10 mg/mL) was used as the positive control for the antifungal assays. The negative control was the respective sterile medium only.

### 2.4. Molecular Identification of Selected Isolates

For molecular identification based on the 16S rRNA gene, the target fragment was amplified by PCR using the primer pair 27F (5′-AGAGTTTGATCCTGGCTCAG-3′) and 1492R (5′-GGTTACCTTGTTACGACTT-3′), according to the method described by Vingataramin et al. [19]. Briefly, selected isolates were cultured in the corresponding isolation broth overnight. Subsequently, 2 mL of each culture was collected and centrifuged at 8000 rpm for 10 min to obtain the cell pellet. The pellet was washed twice with PBS (phosphate buffer containing 0.9% NaCl). After washing, the cell pellet was resuspended in 455 µL of EtNa solution (240 mM NaOH, 74% ethanol, and 2.7 mM EDTA), incubated at 80 °C for 10 min, and centrifuged at 12,000 rpm for 10 min. The pellet was then resuspended in 100 µL DNA suspension buffer (Qiagen, Germantown, MD, USA), and 1.5 µL of the extracted DNA was used as a template for PCR. PCR amplification was then carried out using a PCR Thermocycler (Eppendorf, Enfield, CT, USA) in a total reaction volume containing 12.5 µL of Taq PCR Master Mix, 1 µL of primer 27F, 1 µL of primer 1492R, 1 µL of DNA template, and 9 µL of nuclease-free water. The thermal cycling conditions were as follows: initial denaturation at 96 °C for 3 min; 35 cycles of 95 °C for 45 s, 55 °C for 45 s, and 68 °C for 2 min; followed by final extension at 68 °C for 7 min.

For *Bacillus* sp. isolates, whose 16S rRNA gene sequences provided insufficient resolution for reliable species-level identification, the housekeeping gene *rpoB* was additionally analyzed to improve taxonomic discrimination among closely related taxa. The *rpoB* gene was amplified from the same genomic DNA preparations using the primer pair *rpoB*1206 (5′-ATCGAAACGCCTGAAGGTCCAAACAT-3′) and *rpoB*3202 (5′-ACACCCTTGTTACCGTGACGACC-3′) [20]. Each 25 µL reaction contained 12.5 µL of Taq PCR Master Mix, 1 µL of each primer, 1.5 µL of DNA template, and 9 µL of nuclease-free water. The thermal cycling conditions consisted of initial denaturation at 95 °C for 3 min, followed by 35 cycles of denaturation at 95 °C for 20 s, annealing at 55 °C for 33 s, and extension at 72 °C for 90 s, with final extension at 72 °C for 5 min.

The obtained 16S rRNA and *rpoB* gene sequences were deposited in the National Centre for Biotechnology Information (NCBI) GenBank database (with their accession numbers presented in Tables S3 and S4) before they were edited and assembled by using BioEdit [21]. The consensus sequences were compared with those available in the NCBI GenBank database using blastn algorithm to determine the closest related species. Phylogenetic analysis based on the sequences was performed using MEGA12 with branch support evaluated using 1000 bootstrap replicates to support species-level identification of the isolates [22].

### 2.5. Extraction of the Secreted Compounds from the Culture of a Selected Isolate

Each selected isolate was cultured in 1% LB broth at 30 °C and 200 rpm. A seed culture was first prepared for 12 h, and 10% (*v*/*v*) of the seed culture was then inoculated into fresh medium and incubated for 72 h to obtain fermentation broth enriched with extracellular metabolites. Following centrifugation at 6000 rpm for 15 min at 4 °C, the cell-free supernatants were collected and separately extracted with n-hexane, ethyl acetate, and dichloromethane using a 1:1 (*v*/*v*) solvent-to-supernatant ratio. The mixtures were stirred for 30 min and centrifuged again under the same conditions to achieve clear phase separation. The culture supernatant was also extracted with methanol according to the following procedure: It was passed through a D101 macroporous adsorption resin column to retain extracellular metabolites and subsequently washed with distilled water to remove residual medium components and impurities, after which the adsorbed metabolites were eluted with methanol. Each solvent fraction was collected individually and removed under reduced pressure at 55 °C to yield crude extracts. The crude extracts were dissolved in 10% DMSO to a concentration of about 100 mg/mL and stored at 4 °C until use.

### 2.6. Fractionation of Active Crude Extracts by Column Chromatography

The EtOAc extract was fractionated by silica gel column chromatography using silica gel 60 (70–230 mesh, 0.063–0.200 mm) packed in a glass column (2.5 × 40 cm, internal diameter × length). The column was equilibrated for 2–3 h with n-hexane–acetone (50:1, *v*/*v*) prior to sample loading. Elution was performed using a stepwise n-hexane–acetone gradient with progressively increasing proportions of acetone, followed by a DCM–MeOH system to elute more strongly retained constituents. Eleven consecutive eluates of 20 mL each were collected. The eluates were analyzed by thin-layer chromatography (TLC) on silica gel 60 F254 plates using n-hexane–acetone (2:1, *v*/*v*) as the mobile phase. TLC spots were visualized under UV light at 365 nm. Eluates exhibiting similar TLC profiles, based on the number and relative migration positions of the detected spots, were pooled (to obtain four fractions, designated as F1–F4). They were subsequently tested for antimicrobial activity by using the paper disk diffusion method described below for the crude extracts.

### 2.7. LC–HRMS Characterization of Active Fractions

The LC–HRMS analysis of a sample was conducted in accordance with the previous study by Nguyen et al. [23]. Phytochemical profiling of the active fractions was performed on a Thermo Scientific Ultimate 3000 UPLC system (Thermo Fisher Scientific, Waltham, MA, USA) coupled to a high-resolution Q Exactive mass spectrometer (Thermo Fisher Scientific, Waltham, MA, USA). Chromatographic separation was achieved using a Waters ACQUITY UPLC BEH C18 column (150 mm × 2.1 mm, 1.7 µm particle size) (Waters Corporation, Milford, MA, USA) maintained at a constant temperature of 40 °C. The mobile phase consisted of 0.1% (*v*/*v*) aqueous formic acid (phase B) and 0.1% (*v*/*v*) formic acid in acetonitrile (phase A), delivered at a constant flow rate of 0.2 mL/min with an injection volume of 10 µL. The linear elution gradient was programmed as follows: 0–1.0 min, isocratic at 95% B; 1.0–16.0 min, ramped down from 95% to 0.5% B; 16.0–26.0 min, held at 0.5% B; 26.0–26.2 min, returned to 95% B; and 26.2–30.0 min, re-equilibrated at 95% B. Mass spectra were acquired across an *m*/*z* scan range of 100–1000 in both electrospray ionization (ESI) positive and negative polarities. Full MS scans and data-dependent tandem mass spectrometry (dd-MS^2^) were operated at resolving powers of 70,000 and 17,500 FWHM (at *m*/*z* 200), respectively. The top five most abundant precursor ions per cycle were selected for fragmentation with a normalized collision energy (NCE) set to 40. The raw LC–HRMS datasets were initially obtained in the instrument-specific raw format and subsequently converted to mzML files using MSConvert and ProteoWizard softwares (ProteoWizard software package, version 3.0). The converted datasets were subsequently uploaded to the GNPS2 platform for spectral matching and molecular networking analysis. The corresponding GNPS2 task IDs were as follows: f958ddc731724e1a8bf5e62c27728b5b (positive mode) and 2640e5582fce46889f074c75dc0965f7 (negative mode), collected on 26 and 27 May 2026. Molecular networking was performed using a cosine similarity threshold of ≥0.7, with a minimum of six matched fragment ions required for spectral alignment.

Compound annotation was achieved through MS/MS spectral matching using spectral library searching based on publicly available reference libraries, including GNPS, ENAMDISK, MCE Bioactive, NIH Natural Products, and MassBank. Spectral matching was performed for both positive and negative ionization datasets using precursor and fragment ion mass tolerances of 0.01 Da (10 ppm). Putative identifications were accepted when the matched spectra contained at least six corresponding fragment ions and achieved a weighted cosine similarity score of ≥0.70.

Based on the criteria established by the Metabolomics Standards Initiative (MSI), the annotated metabolites were assigned as putatively identified compounds (MSI Level 2). These assignments were supported by accurate precursor mass values, agreement with reference MS/MS spectral data, and characteristic fragmentation patterns.

### 2.8. Agar Diffusion Assay to Evaluate the Antimicrobial Activity of a Crude Extract or a Chemical Fraction

The antimicrobial activity of a crude extract or a chemical fraction of interest was evaluated at different concentrations by using the paper disk diffusion method. Sterile paper disks (5 mm in diameter) were loaded with the tested solution and placed onto agar plates previously inoculated with the corresponding test microorganisms, and 10 µL of each test solution was loaded onto each paper disk. A solvent control containing 10 µL of 10% DMSO was included as the negative control. Erythromycin (10 mg/mL) was used as the positive control for the antibacterial assays against *B. subtilis* and *X. oryzae*, whereas salicylic acid (10 mg/mL) was used as the positive control for the antifungal assays against *F. oxysporum* and *P. asparagi*. The negative control was the respective sterile medium only. After incubation under the appropriate conditions for each test microorganism, antimicrobial activity was assessed by measuring the diameter of the inhibition zone around each disk.

### 2.9. Minimum Inhibitory and Minimum Bactericidal Concentration

The minimum inhibitory concentration of an active crude extract was determined by means of a microdilution assay in sterile 96-well microplates following Clinical and Laboratory Standards Institute (CLSI)-based procedures [24]. The bacterial indicator suspensions were standardized to approximately 1 × 10^6^ CFU/mL before use. Crude extracts were initially dissolved in DMSO and the resulting solutions were further diluted with water to obtain final tested concentrations of 100, 50, 25, 12.5, 6.25, 3.13, 1.56 and 0.78 mg/mL. For the highest extract concentration (100 mg/mL), the corresponding concentration of DMSO present in the solution was 10% (*v*/*v*); thus, a 10% (*v*/*v*) DMSO control without extract was included as the negative solvent control. Erythromycin (10 mg/mL) was used as the positive antibacterial control. After incubation at 30 °C for 24 h, the MIC was defined as the lowest extract concentration at which no visible microbial growth was observed. For MBC determination, aliquots from wells showing no visible growth were spread onto freshly prepared agar plates and incubated again. The lowest concentration resulting in complete killing of the test microorganism was recorded as the MBC. All experiments were carried out in triplicate.

### 2.10. Statistical Analysis

All quantitative antimicrobial assays were performed in triplicate, and the experiments were repeated at least twice. Data are presented as the mean ± standard deviation (SD). For the primary antimicrobial screening, differences in inhibition zone diameters among isolates were analyzed separately for each test microorganism using one-way analysis of variance (ANOVA), followed by Tukey’s multiple-comparison test. Homogeneity of variances was assessed using the Brown–Forsythe test. For crude extract assays involving both isolate and extraction solvent effects, two-way ANOVA followed by Tukey’s multiple-comparison test was applied where appropriate. Statistical significance was accepted at *p* < 0.05. All statistical analyses and graphical presentations were performed using GraphPad Prism 10 (GraphPad Software, Boston, MA, USA).

## 3. Results

### 3.1. Isolation of Seaweed-Associated Microorganisms

**Figure 1:**
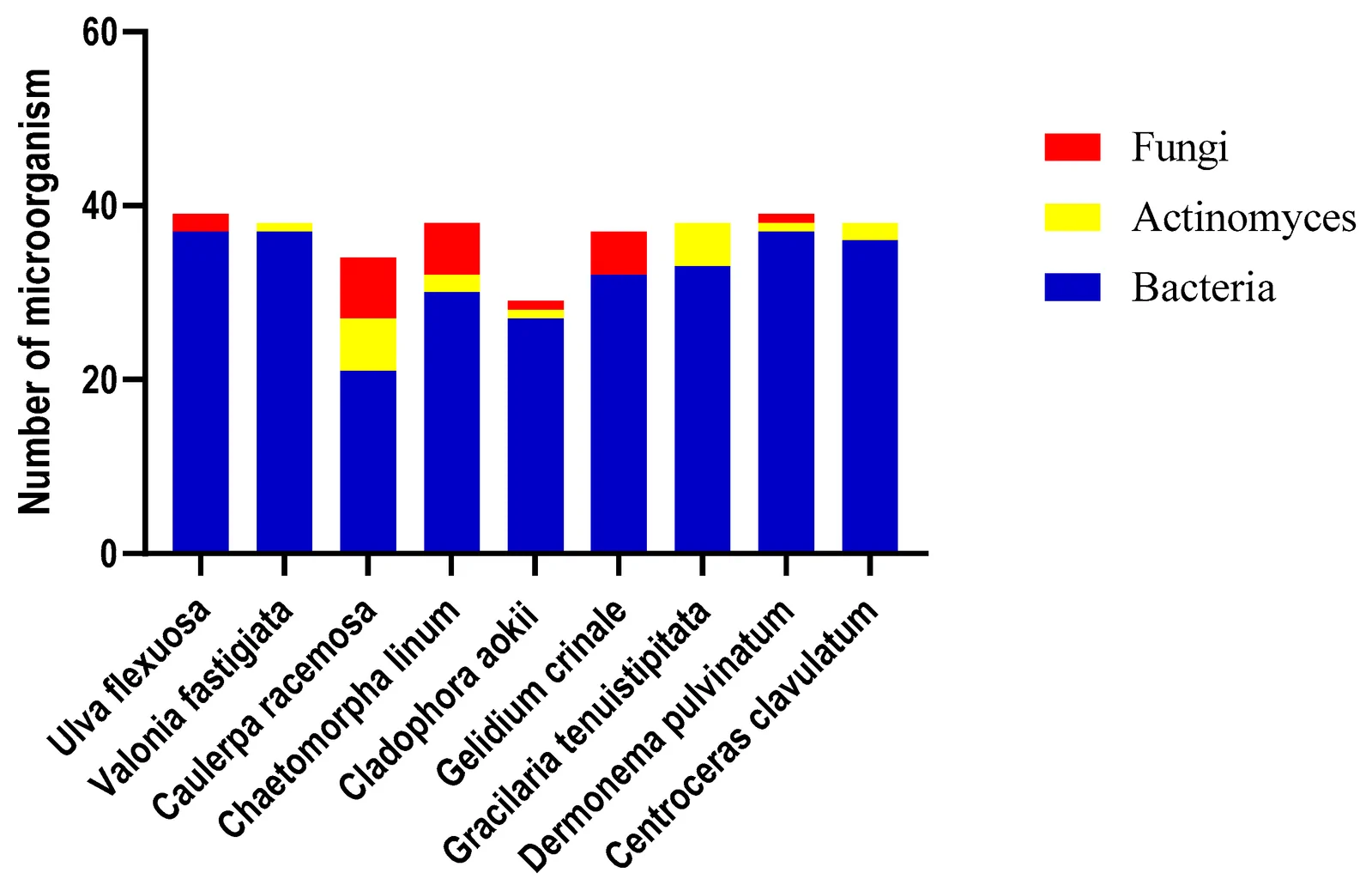
Distribution of culturable microorganisms isolated from different macroalgal hosts.

**Figure 2:**
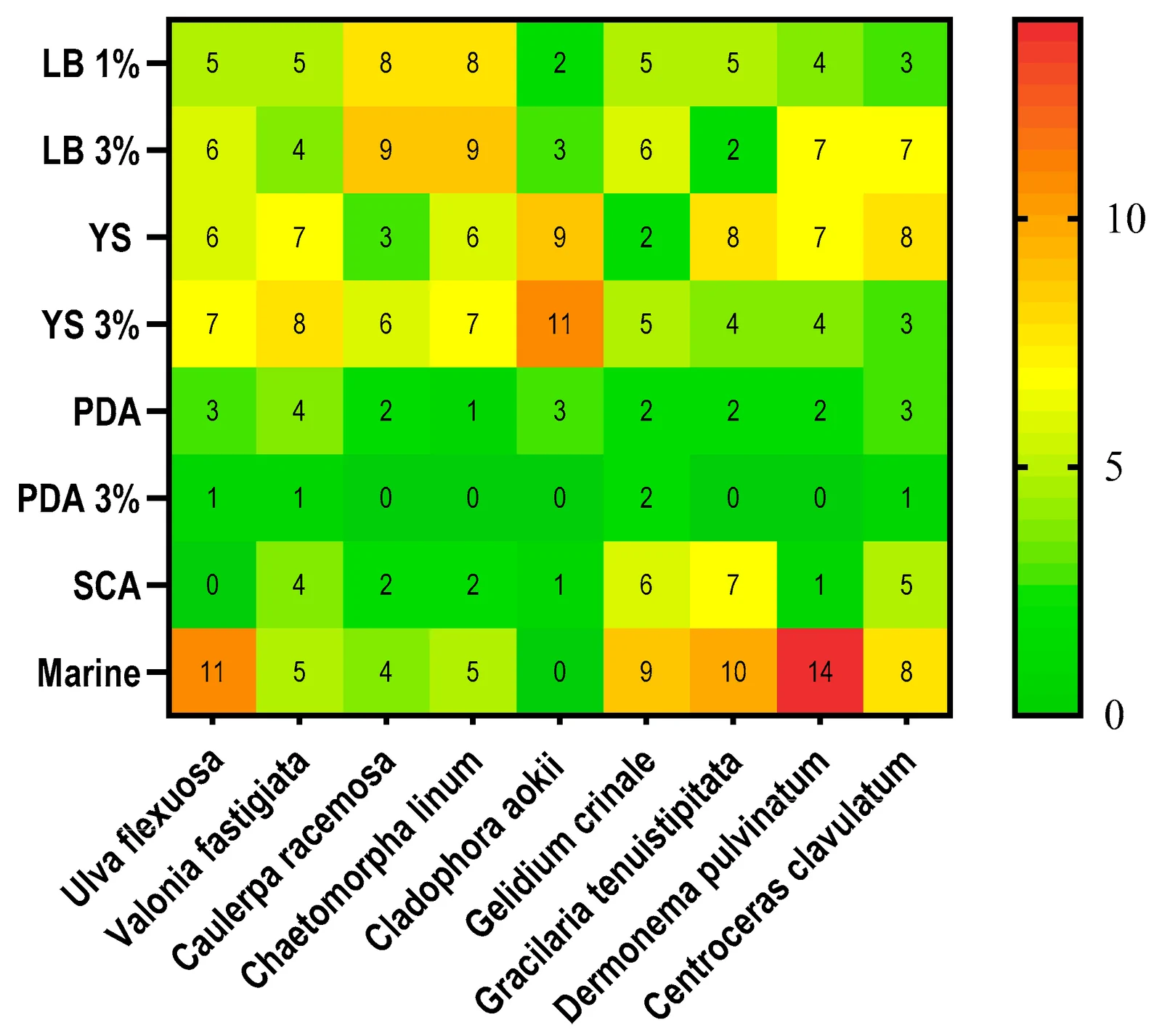
Heatmap of isolate recovery across eight culture media and nine macroalgal hosts.

A total of 331 culturable seaweed-associated epiphytic and endophytic isolates were recovered from nine seaweed hosts collected from Cat Ba Archipelago, Hai Phong, Vietnam. *Gracilaria tenuistipitata* was the seaweed with the highest number of isolates obtained (43 isolates), followed by *Ulva flexuosa*, *Dermonema pulvinatum* (39 isolates), *Valonia fastigiata* (38 isolates), *Chaetomorpha linum*, *Gelidium crinale* (37 isolates), *Centroceras clavulatum* (36 isolates), *Caulerpa racemosa* (33 isolates), and *Cladophora aokii* (29 isolates) (Figure 1). Across all these hosts, bacterial isolates predominated, whereas actinomycetous and fungal isolates accounted for smaller proportions of the total number of isolates. The recovery of isolates also varied among the eight isolation media, with Marine, LB 3%, and YS-based media generally yielding more isolates than PDA-based media, suggesting that both host identity and culture conditions influenced the culturability of seaweed-associated microorganisms (Figure 2).

### 3.2. Screening for Antimicrobial Activity

The obtained isolates were screened for antimicrobial activity. A total of fifteen seaweed-associated bacterial isolates exhibited detectable antimicrobial activity against at least one of the four test microorganisms (Table 1 and Table S2 and Figure S1). Among the test microorganisms, *Bacillus subtilis* was the most susceptible target, as all 15 isolates showed inhibitory activity against this test microorganism, whereas 10 isolates were active against *Xanthomonas oryzae* sp. *oryzae*, 10 isolates against *Fusarium oxysporum*, and 6 isolates against *Phomopsis asparagi*.

Several isolates displayed broad-spectrum antimicrobial activity. In particular, S.002, S.323, S.374, S.467, S.556, and S.593 inhibited all four test microorganisms, whereas the remaining isolates were active against one to three targets only. Among these, S.002 and S.556 showed the strongest activities against both test bacteria and fungi with the broadest spectra. In addition, S.002 and S.556 exhibited strong activity against *X. oryzae* together with moderate activity against the other test microorganisms. Based on their overall antimicrobial performances, S.002, S.374, S.556, and S.593 were selected for subsequent characterization and further experiments.

### 3.3. Identification Based on Morphological and Phylogenetic Analysis of the Isolates with Outstanding Antimicrobial Activities

**Figure 3:**
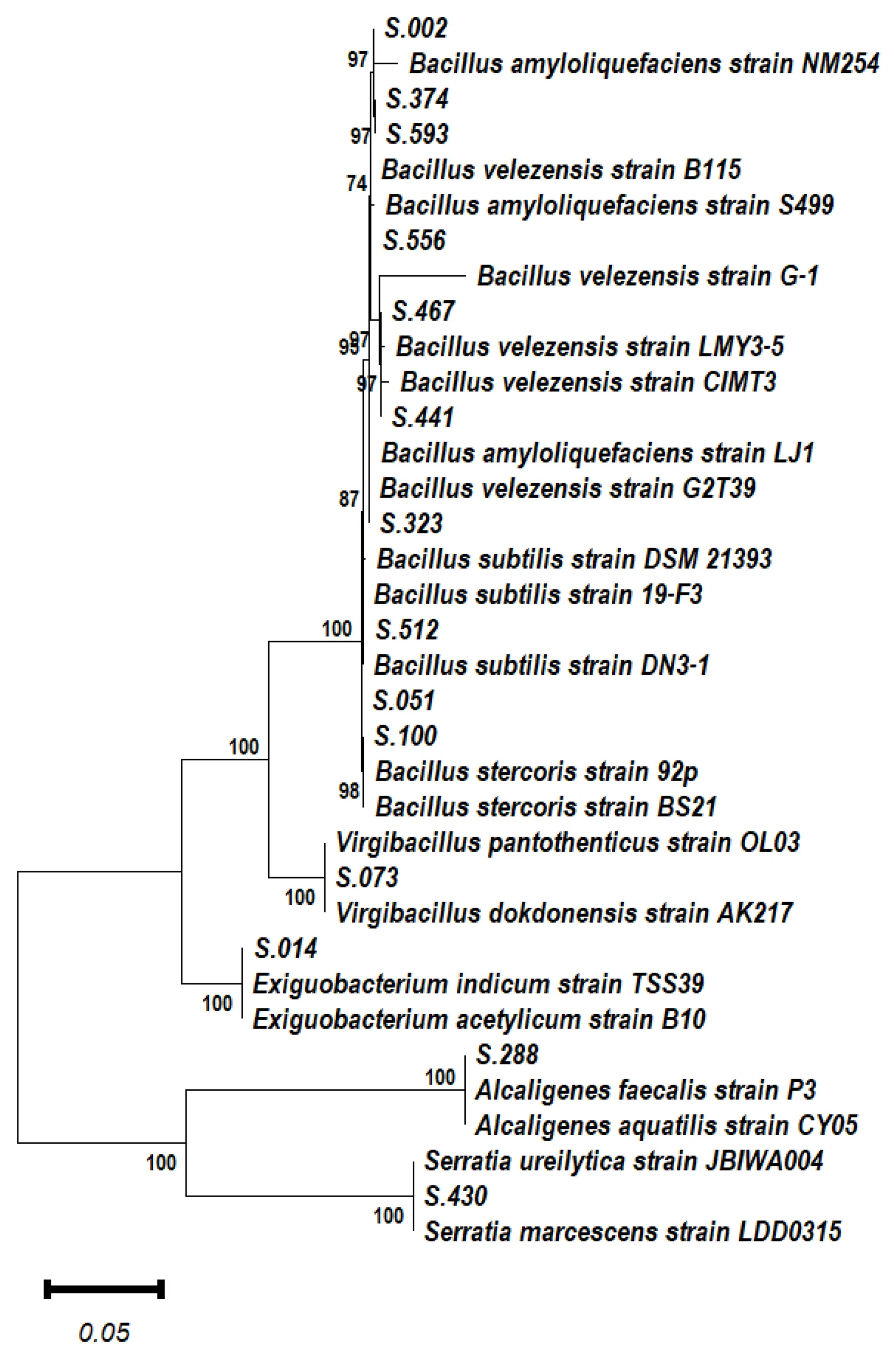
Phylogenetic tree based on 16S rRNA gene sequences of bacterial isolates with outstanding antimicrobial activities.

**Figure 4:**
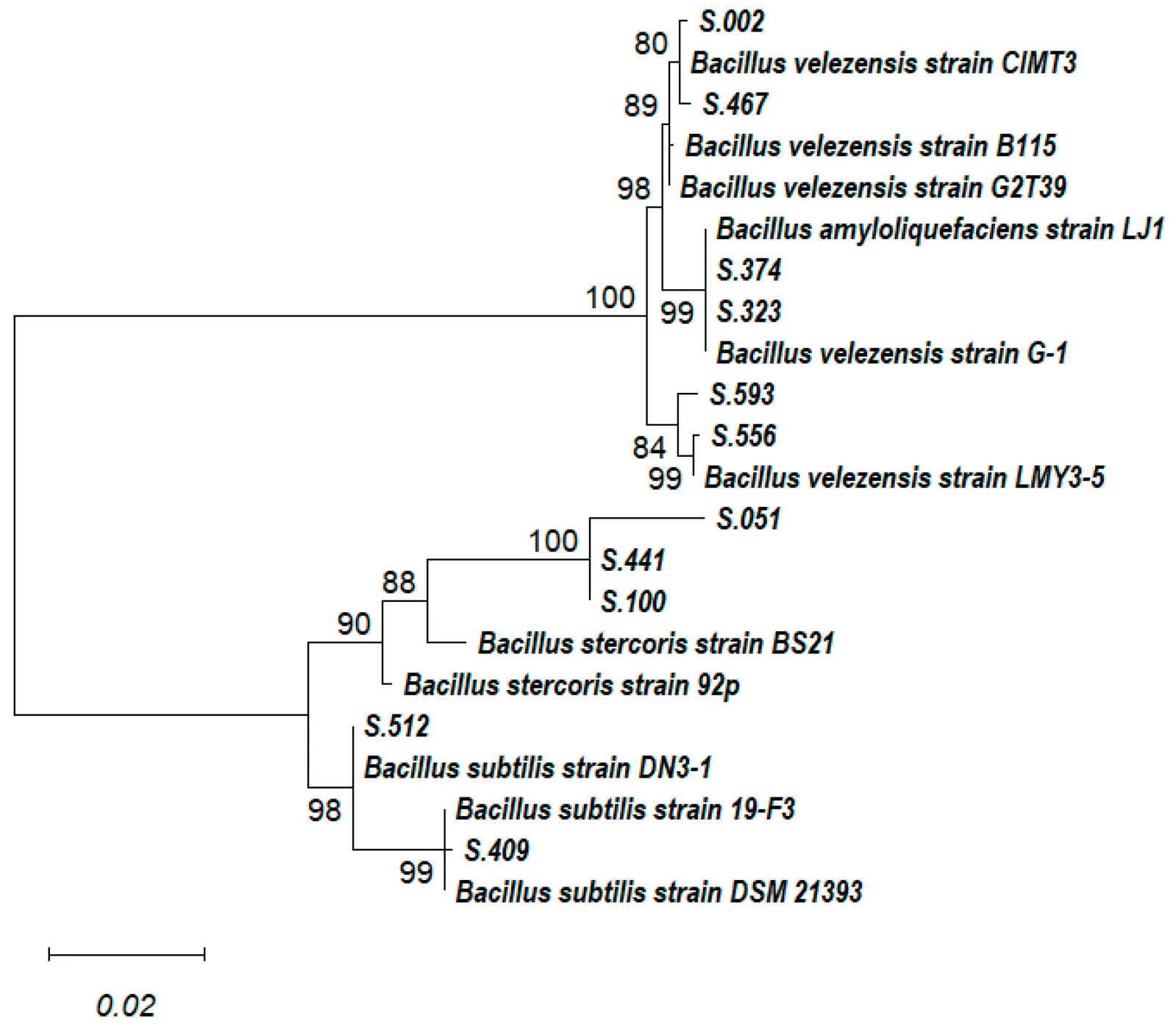
Phylogenetic relationships of bioactive Bacillus isolates based on partial *rpoB* gene sequences.

Morphological characterization (Figure S2) together with 16S rRNA gene sequence analysis (Figure 3) revealed that the 15 bacterial isolates with noticeable antimicrobial activities were affiliated with five bacterial genera. Specifically, eleven isolates are all Gram (+) with rod-shaped cells and their 16S rRNA genes have >99% homology to those of *Bacillus* species. Based on *rpoB*-based phylogeny analysis (Figure 4, Table S4), they were further identified as belonging to *Bacillus* species: S.002, S.467, S.556 and S.593 as *B. velezensis*; S.374 and S.323 as *B. amyloliquefaciens*; S.051, S.441, and S.100 as *B. stercoris*; and S.512 and S.409 as *B. subtilis*, with >97% sequence homology in all cases. The remaining four isolates were identified to belong to several genera outside the *Bacillus* clade, i.e., *Virgibacillus*, *Alcaligenes*, *Exiguobacterium* and *Serratia*-related reference strains. The results indicate that *Bacillus*-related bacteria were a predominant source of seaweed-associated microorganisms with antimicrobial activities.

### 3.4. The Antimicrobial Activities of the Crude Extracts of the Cultures of the Selected Isolates

Crude extracts obtained from the selected isolates S.002, S.374, S.556, and S.593 using n-hexane, ethyl acetate (EtOAc), dichloromethane (DCM), and methanol (MeOH) exhibited markedly different antimicrobial profiles (Table 2). No detectable activity was observed for any of the n-hexane extracts. In contrast, EtOAc, DCM, and MeOH extracts showed antibacterial activity against *B. subtilis* and *X. oryzae*, although the magnitude of inhibition varied according to both the producing isolate and extraction procedure. Against *B. subtilis*, the S.002 EtOAc extract produced the largest inhibition zone (11.50 ± 0.20 mm in diameter), followed closely by the S.002 and S.556 MeOH/DCM extracts (10.00–11.00 mm). A more pronounced inhibitory effect was generally observed against *X. oryzae*. The strongest activity was recorded for the S.002 EtOAc extract (with the inhibition zone diameter of 23.50 ± 0.30 mm), although this was not significantly different from the S.002 and S.556 MeOH extracts (with the inhibition zone diameters of ~23.00 ± 0.20 mm; *p* > 0.05). DCM extracts of S.002 and S.556 also showed substantial activity, with inhibition zone diameters of 22.00 ± 0.20 and 21.50 ± 0.40 mm, respectively. Overall, the antibacterial activity showed a clear isolate- and extraction-dependent pattern.

A distinct pattern was observed for the fungal targets. Whereas the n-hexane, EtOAc, and DCM extracts showed no detectable inhibition of *F. oxysporum* or *P. asparagi*, antifungal activity was consistently recovered in the MeOH extracts of all four isolates. The S.002 MeOH extract exhibited the greatest antifungal activity, with inhibition zone diameters of 13.00 ± 0.20 mm against *F. oxysporum* and 14.00 ± 0.30 mm against *P. asparagi*. This was followed by S.374 (12.00 ± 0.10 and 13.00 ± 0.10 mm, respectively), whereas S.556 and S.593 showed comparatively lower but detectable antifungal activity. These results demonstrate that the antimicrobial spectrum of the recovered crude extracts was strongly dependent on the extraction procedure: EtOAc and DCM preferentially recovered antibacterial constituents, whereas the D101 adsorption–MeOH elution procedure additionally recovered constituents associated with antifungal activity.

### 3.5. Minimum Inhibitory and Bactericidal Concentrations of Active Crude Extracts

The antibacterial activity of the EtOAc, DCM and MeOH extracts was further evaluated by determining their minimum inhibitory concentrations (MICs) and minimum bactericidal concentrations (MBCs) against *B. subtilis* and *X. oryzae* (Table 3).

Among the EtOAc extracts, S.002 exhibited the most consistent activity against both the test organisms, with an MIC of 6.25 mg/mL and an MBC of 25 mg/mL. The S.374 EtOAc extract showed comparable activity against *B. subtilis* but was substantially less active against *X. oryzae*, with MIC and MBC values of 50 and 100 mg/mL, respectively. The EtOAc extracts of S.556 displayed relatively weak activity against both bacteria, whereas those of S.593 were more active against *X. oryzae* than against *B. subtilis*, with corresponding MIC values of 12.5 and 100 mg/mL.

For the DCM extracts, S.002 again displayed the lowest MIC against both the test organisms at 12.5 mg/mL. Its MBC values were 50 mg/mL against *B. subtilis* and 25 mg/mL against *X. oryzae*. In contrast, the remaining DCM extracts showed MIC values ranging from 25 to 50 mg/mL and generally required 100 mg/mL to achieve bactericidal activity. Differences between EtOAc and DCM were dependent on both the producing isolate and the target bacterium, indicating that neither solvent consistently recovered the most active constituents from all four isolates.

The MeOH extracts generally exhibited lower antibacterial potency than the corresponding EtOAc and DCM extracts. S.002 and S.374 showed the greatest activity among the MeOH extracts, with MIC values of 25 mg/mL against *B. subtilis*. Against *X. oryzae*, S.002 also exhibited an MIC of 25 mg/mL, whereas S.374 showed a higher MIC of 50 mg/mL. The S.556 MeOH extract displayed an MIC of 50 mg/mL against both test bacteria, while S.593 showed the weakest activity against *B. subtilis* at 100 mg/mL but greater activity against *X. oryzae* at 50 mg/mL. All MeOH extracts required 100 mg/mL to achieve bactericidal activity against both organisms. Overall, these findings indicate that MeOH recovered antibacterial constituents, although their activity was generally weaker than that of the EtOAc extracts, particularly for S.002.

The calculated MBC/MIC ratios ranged from 1 to 4. Based on the commonly applied criterion for MBC/MIC ratios, all tested extracts exhibited a bactericidal effect rather than exclusively bacteriostatic effect under the experimental conditions [25]. Nevertheless, such a bactericidal effect classified based on this ratio did not necessarily correspond to high antibacterial activity. For example, the S.593 EtOAc extract has an MBC/MIC ratio of 1 against *B. subtilis*, although both its MIC and MBC were relatively high at 100 mg/mL. Overall, S.002 EtOAc demonstrated the most remarkable and consistent antibacterial profile, with the lowest MIC values against both target bacteria and an MBC of 25 mg/mL.

Given its stronger and more reproducible antimicrobial activity compared with those of the other selected strains, S.002 was prioritized for subsequent chemical investigation. Another reason for this consideration is that S.002 represents a *Bacillus velezensis* strain associated with *Gelidium crinale*, a comparatively underexplored red macroalga. Considering that EtOAc displayed the highest efficiency in recovering the compounds in the S.002 culture, we used the EtOAc extract of S.002 for further chemical characterization.

### 3.6. The Antimicrobial Activities of the Fractions Obtained from the Crude Extract of S.002 Culture

The EtOAc crude extract of S.002 was subjected to silica gel column chromatography, yielding 11 eluate tubes that were pooled into four fractions (F1–F4) according to TLC similarity. Fraction F1 was obtained from the early n-hexane/acetone eluates, fraction F2 from the later HA and initial DM eluates, fraction F3 from the intermediate DM eluates, and fraction F4 from the final methanol eluate.

The resulting fractions were evaluated for antibacterial activity against *B. subtilis* and *X. oryzae*. Among the four fractions obtained, only fraction F1 and fraction F4 showed detectable antibacterial activity. Specifically, fraction 1 inhibited *B. subtilis* (with an inhibition zone diameter of 16.5 ± 0.2 mm), whereas fraction F4 was active against *X. oryzae* (with an inhibition zone diameter of 19.0 ± 0.3 mm) (Table 4 and Figure S3). In contrast, fractions F2 and F3 showed no detectable activity against either indicator strain. These results indicate that the antibacterial activity of the EtOAc extract was attributed to certain fractions rather than being evenly distributed throughout the whole extract.

### 3.7. LC–HRMS Characterization of Active Fractions

As fraction F4 displayed the most pronounced activity and such activity was against *X. oryzae*, a rice pathogen of great interest in Vietnam, it was selected for LC–HRMS analysis to investigate its chemical composition. LC–HRMS profiling tentatively identified 24 compounds in this fraction (Table 5 and Table S5), including representatives of several primary and secondary metabolites. These compounds belong to several groups, such as cyclic dipeptides/diketopiperazines (Cyclo(L-Phe-D-Pro); Cyclo(-His-Pro); Cyclo(proline-leucine); Cyclo(L-Leu-L-4-Hyp); Cyclo(Phe-Leu), and PyroGlu-Pro), cyclic lipopeptides (Surfactin C; Surfactin B; Bacillomycin LC2, and Surfactin A), lipoamino acids (N-tetradecanoylthreonine, N-hexadecanoylserine, N-hexadecanoylthreonine, N-pentadecenoylleucine, N-pentadecanoylserine, N-hexadecenoylserine, N-octadecenoylserine, N-hexadecenoylthreonine, and N-octadecadienoylserine), oxidized lipoamino acids (3-hydroxyhexadecanoyl threonine and 3-hydroxyhexadecanoyl serine), alkaloid-like metabolites (Norharmane and Quinoline-4-carboxylic acid), and an amino acid derivative (Tryptophan).

Among these, the cyclic lipopeptides Surfactin A, Surfactin B, Surfactin C, and Bacillomycin LC2 were particularly noteworthy because they belong to metabolite classes commonly associated with antimicrobial activity in *Bacillus* species. The presence of several diketopiperazines and amphiphilic lipoamino acids further suggests that this active fraction contains multiple classes of bioactive microbial metabolites. Taken together, these findings indicate that the biological activity of the active fraction may result from a chemically diverse mixture of peptide-derived and lipid-associated metabolites rather than from a single compound alone.

## 4. Discussion

The recovery of 331 culturable microorganisms from nine seaweed hosts suggests that marine macroalgae from the Cat Ba Archipelago area represent a diverse reservoir of cultivable host-associated microbiota. In terms of both host coverage and the number of isolates, this collection exceeds those reported in several earlier culture-dependent surveys. Ismail et al. (2016), for example, obtained 26 initial bacterial isolates from the brown alga *Padina pavonica*, of which 18 morphologically distinct cultures were retained, whereas Karthick et al. (2018) recovered 77 epiphytic bacterial isolates from eight intertidal seaweed species [35,36]. Direct numerical comparisons among studies should nevertheless be made cautiously because isolate recovery is strongly affected by sampling effort, tissue pretreatment, dilution strategy, medium composition, salinity, incubation temperature and colony selection criteria. Thus, the relatively large collection obtained here suggests that the Cat Ba Archipelago area is an attractive place to study seaweed and seaweed-associated microorganisms with potential bioactivities.

The number of recovered isolates varied markedly among the sampled hosts, with *Gracilaria tenuistipitata* yielding the largest collection and *Cladophora aokii* the smallest. Such variation suggests that host identity influences the fraction of associated microorganisms that can be cultivated under a given set of laboratory conditions. Macroalgal surfaces are not passive substrata; they differ in thallus architecture, surface chemistry, nutrient exudation, polysaccharide composition and physiological status, all of which may influence microbial attachment and persistence [12]. These host-related factors act together with local environmental conditions to create distinct microniches on and within algal tissues [37].

One of the key findings of this study is the discovery of the antimicrobial bacteria detected for the first time in their respective hosts (Table 1). Considering each algal host, such novel associations are interesting, as discussed below:

- The red alga *Gelidium crinale* has not been reported to harbor *Bacillus velezensis* and *Exiguobacterium* species. Several culture-based studies have identified the presence of bacteria belonging to diverse genera such as *Sulfitobacter*, *Aquimarina*, *Maribacter*, *Alcaligenes*, *Flavobacterium*, and *Pseudomonas*, solely in the surface of the thallus surface of the alga [38,39]. However, it is surprising that no bacillii have been found in this alga though they are common endophytes. *Bacillus velezensis* is well known for its diverse bioactivities that may substantially support the host, based on its production of specialized cyclic lipopeptides and bacteriocins that prevent biofouling and suppress opportunistic pathogens [40]. Furthermore, the capacity of seaweed-associated *Bacillus velezensis* strains for surface colonization, biofilm formation and osmotic adaptation may favor persistence on the algal thallus and regulation of neighboring microorganisms [41]. *Exiguobacterium* species are less known for their bioactivities, but they may facilitate localized nutrient cycling and protect the host tissue from degradation due to their ability of forming resilient biofilm structures [42].
- The green alga *Ulva flexuosa* has previously been shown to harbor polysaccharide-degrading bacteria such as *Bacillus subtilis* and *Shewanella algae*, which may contribute to the transformation of complex algal or environmental carbohydrates [43]. However, the present study found that *U. flexuosa* harbored *Bacillus stercoris* and *Virgibacillus* species, neither of which was identified as specifically associated with the host. *Bacillus stercoris*, with reported traits including phosphate solubilization, extracellular enzyme production, and microbial antagonism, may definitely play some roles in nutrient mobilization and competitive exclusion for the host [44]. *Virgibacillus* bacteria also exhibit pronounced antimicrobial bioactivities through specialized pathogen-suppressing peptides and even secret diverse secondary metabolites, thereby actively influencing macroalgal phycosphere dynamics [45,46]. These findings therefore broaden the knowledge on the microbiota of *U. flexuosa*. Their antimicrobial activities suggest that the bacteria associated with this alga may perform complementary ecological functions, ranging from polysaccharide turnover to interference with competing surface colonizers.
- Similar functional complementation was also evident when looking at the bacteria associated with the red alga *Gracilaria tenuistipitata*. The main previous finding was the isolation of the agarolytic bacterium *Flavobacterium* sp. ARAG 55.4 from this host, highlighting the contribution of associated bacteria to the degradation and recycling of sulfated algal polysaccharides [47]. In the present study, *Bacillus stercoris*, *Bacillus velezensis*, and *Alcaligenes* sp. were identified as antimicrobial members of the same host-associated microbiota, suggesting their ecological functions beyond polysaccharide turnover. The roles of *Bacillus stercoris* and *Bacillus velezensis* may be similar to those with other algae, as discussed above. Regarding *Alcaligenes* species, these are known to produce vitamin B_12_ and mobilize poorly available phosphate, suggesting a potential contribution to nutrient exchange within the phycosphere. Collectively, these bacteria may form a functionally complementary assemblage involved in nutrient recycling, micronutrient provisioning, surface colonization, and microbial antagonism [48,49], which certainly benefit the host *G. tenuistipitata*.
- A similar novel host–microbe functional relationship was also observed for the alga *Chaetomorpha linum*. The discovery of *Bacillus subtilis, Bacillus stercoris* and *Serratia* sp. expands both the taxonomic and functional diversity currently documented for the cultivable associated microorganisms of *Chaetomorpha linum*. Previous culture-based studies recovered carbohydrase-producing *Bacillus* isolates from *Chaetomorpha* sp.; however, neither the algal host nor the bacterial isolates were resolved to the species level [50]. The present study therefore is the first to identify antimicrobial *Bacillus subtilis* specifically associated with *C. linum*. Noticeably, high-throughput profiling of the epiphytic microbiome of this alga revealed a community dominated primarily by bacteria belonging to *Leucothrix*, *Algimonas*, *Hellea*, *Granulosicoccus*, and *Dokdonia*, whereas neither *Bacillus* nor *Serratia* was reported among the dominant taxa [51]. The recovery of a *Serratia* sp. is particularly noteworthy because *Serratia* are commonly known as pathogens to marine organisms but recently there has been some evidence that marine *Serratia* bacteria isolated from another macroalgal host can exhibit antimicrobial activity, solubilize phosphate and produce siderophores and indole-3-acetic acid, suggesting their potential roles to the host through complementary processes involving microbial antagonism, nutrient exchange, and community regulation [52].
- For *Centroceras clavulatum* and *Caulerpa racemosa*, the fact that the associations with *Bacillus amyloliquefaciens* and *Bacillus subtilis* were found for the first time is also surprising because the association of *Bacillus* species with other organisms is common. For *C. clavulatum*, previous studies obtained several isolates exhibiting antibacterial and anticancer properties from it, but did not taxonomically identify them [53]. The identification of *Bacillus amyloliquefaciens* therefore provides a more defined phylogenetic context for the bioactive microbiota of this red alga. Like *B. velezensis*, *B. amyloliquefaciens* can produce structurally diverse antimicrobial metabolites, including lipopeptides, polyketides, and other antibacterial compounds, which probably benefit the host [54,55]. For *C. racemosa*, earlier work recovered 11 cultivable bacterial isolates from it, with the most active representatives assigned mainly to *Vibrio*, *Salinovibrio*, and related *Vibrionaceae* lineages [56]. The recovery of *Bacillus subtilis*, commonly known antimicrobial agents, in the present study expands this host-associated bioactive repertoire.
- Particularly noteworthy were the discovery of bacteria associated with *Valonia fastigiata*, *Cladophora aokii*, and *Dermonema pulvinatum*, for which host-specific microbiological information remains extremely limited. Within the literature published to date, there have been no prior reports on the association of *B. velezensis* with *V. fastigiata* or of *B. amyloliquefaciens* with *C. aokii* and *D. pulvinata.* Therefore, the identification of these *Bacillus* speices, represented by isolates S.374, S.467, and S.593, in these two algae is interesting and substantially extends the bacterial symbiont records of these underexplored macroalgae. The outstanding bioactivities of *B. velezensis* and *B. amyloliquefaciens*, as discussed above, must definitely have complementary functions related to nutrient transformation, surface colonization, community regulation, and antimicrobial defense that benefit the hosts.

It should be noted that, in this study, due to limited resources, we only investigated microorganisms on single algal samples, and thus, the commonness of the discovered associations may require confirmation in further studies.

Among the active isolates, strain S.002, assigned to the *Bacillus velezensis* species, displayed the most pronounced antimicrobial phenotype. The retention of inhibitory activity in the ethyl acetate extract indicates that the effect was mediated, at least in part, by extracellular metabolites. Members of *B. velezensis* possess extensive biosynthetic capacities and are known to produce cyclic lipopeptides, including surfactins, iturins, bacillomycins and fengycins, together with polyketides, dipeptide antibiotics and siderophores [57,58]. In addition to these canonical lipopeptides, S.002 can produce a number of other metabolites that have merely been reported in chemical studies of *Bacillus velezensis*, including some alkaloids (norharmane and quinoline-4-carboxylic acid) and a unique bioactive amino acid (D-tryptophan), as revealed by LC–HRMS profiling (Table 5). The co-production of these chemically distinct metabolites is therefore likely to broaden and reinforce the antimicrobial phenotype of S.002 beyond that expected from lipopeptides (e.g., surfactins) alone. This is because norharmane, quinoline-4-carboxylic acid and D-tryptophan themselves have antibacterial, antibiofilm or antivirulence activities, which can synergistically enhance the antimicrobial efficacy of S.002, primarily owing to its lipopeptides. Thus, the distinguishing feature of S.002 lies not simply in the production of characteristic *B. velezensis* lipopeptides, but in their integration within a substantially more diverse extracellular metabolite assemblage.

The macroalgal origin of strain S.002 adds an ecologically relevant dimension to this chemical phenotype. Macroalgal thalli form dynamic interfaces characterized by fluctuating salinity, oxidative stress, intense competition for space and continuous exposure to host-derived polysaccharides and low-molecular-weight compounds [59]. Within such environments, secondary metabolites may fulfill more than one function. Amphiphilic lipopeptides can influence surface motility, attachment and biofilm organization, whereas siderophores and diffusible antimicrobial compounds may enhance nutrient acquisition and suppress competitors [60]. Previous studies have likewise reported broad antibacterial activity from macroalga-associated *B. velezensis* strains, with some effects linked to siderophore production or extracellular secondary metabolites [40,61]. The importance of S.002 therefore lies not yet in the demonstration of chemical novelty, but in the identification of a strongly bioactive and chemically tractable strain from an underexplored macroalgal habitat in northern Vietnam. More importantly, it is highly possible that the bacterium may greatly contribute to the overall bioactivity of the algae *Gelidium crinale* to survive in a competitive environment, such as the ocean. *B. velezensis* strain S.002 therefore deserves further investigation, which should include evaluation against a broader panel of clinically or industrially relevant microorganisms. Bioassay-guided sub-fractionation will be required to determine whether the activity arises from a single major compound, several independent metabolites or synergistic interactions among extract components [62]. Further development should also address cytotoxicity toward mammalian cells, selectivity indices, antibiofilm activity, chemical stability, and fermentation yield.

## 5. Conclusions

This study demonstrates that seaweed from the Cat Ba Archipelago area harbors a diverse collection of culturable microorganisms. A total of 331 isolates were recovered from nine macroalgal hosts using media with different nutrient and salinity conditions, highlighting the combined influences of host identity and cultivation strategy on culture efficiency. The isolates displaying notable antimicrobial activities were affiliated predominantly with *Bacillus* species, together with representatives of *Virgibacillus*, *Alcaligenes*, *Exiguobacterium* and *Serratia*, indicating that antimicrobial activity was distributed across several bacterial lineages. Interestingly, almost all of the associations of those bacteria with the seaweed hosts have not yet been reported. Among the selected isolates, *Bacillus velezensis* S.002 exhibited the most consistent antibacterial activity, and its ethylacetate extract showed the lowest MIC values against both *B. subtilis* and *X. oryzae*. Fractionation of the extract yielded fractions that exhibited different antagonistic activities against *B. subtilis* and *X. oryzae*, which suggests the production of various chemically distinct antibacterial metabolites by S.002. Collectively, these findings highlight Cat Ba macroalgae as promising sources of bioactive microorganisms and identify S.002 as a priority candidate for further bioassay-guided isolation and purification of bioactive compounds, together with whole-genome sequencing and biosynthetic gene cluster mining, to elucidate the genetic basis of its antimicrobial potential.

## Figures and Tables

**Table 1:** Antimicrobial activity of selected seaweed-associated bacterial isolates. Note: Highlighted in bold are strains with outstanding activities.

Isolate Code	Seaweed	Microbial Group	Origin	Antimicrobial Activity			
				*B. subtilis*	*X. oryzae*	*F. oxysporum*	*P. asparagi*
**S.002**	*Gelidium crinale*	Bacteria	Endophytic	+++	++++	+++	+++
S.014	*Gelidium crinale*	Bacteria	Endophytic	++	−	−	−
S.051	*Ulva flexuosa*	Bacteria	Epiphytic	+++	++	−	−
S.073	*Ulva flexuosa*	Bacteria	Endophytic	+	++	−	−
S.100	*Gracilaria tenuistipitata*	Bacteria	Endophytic	+++	−	−	−
S.288	*Gracilaria tenuistipitata*	Bacteria	Endophytic	+++	++++	+++	−
S.323	*Centroceras clavulatum*	Bacteria	Epiphytic	++	++	+++	+++
**S.374**	*Valonia fastigiata*	Bacteria	Epiphytic	+++	+++	+++	++++
S.409	*Chaetomorpha linum*	Bacteria	Endophytic	+	−	+++	−
S.430	*Chaetomorpha linum*	Bacteria	Endophytic	+	+	++	−
S.441	*Chaetomorpha linum*	Bacteria	Epiphytic	++	−	−	−
S.467	*Cladophora aokii*	Bacteria	Endophytic	++	++	++	++
S.512	*Caulerpa racemosa*	Bacteria	Epiphytic	++	−	+	−
**S.556**	*Gracilaria tenuistipitata*	Bacteria	Endophytic	+++	++++	++	++++
**S.593**	*Dermonema pulvinatum*	Bacteria	Endophytic	+++	+++	+++	+++

Notes: The antimicrobial activity of each isolate was evaluated against five levels according to the inhibition zone diameter (d): “−”, “+”, “++”, “+++”, and “++++”, corresponding to d = ~5.00 mm, d = less than or equal to 8.00 mm, d = 8.01–11.00 mm, d = 11.01–15.00 mm and d = above 15.00 mm, respectively.

**Table 2:** The antimicrobial activity of the crude extracts of the cultures from the selected isolates obtained with different organic solvents.

Crude Extracts		Diameter of the Inhibition Zone (mm)			
		*Bacillus subtilis*	*Xanthomonas oryzae*	*Fusarium oxysporum*	*Phomopsis asparagi*
S.002	n-hexane	-	-	-	-
	EtOAc	11.50 ± 0.20 ^a^	23.50 ± 0.30 ^a^	-	-
	DCM	10.00 ± 0.20 ^cd^	22.00 ± 0.20 ^b^	-	-
	MeOH	11.00 ± 0.10 ^ab^	23.00 ± 0.20 ^a^	13.00 ± 0.20 ^a^	14.00 ± 0.30 ^a^
S.374	n-hexane	-	-	-	-
	EtOAc	10.00 ± 0.30 ^cd^	16.00 ± 0.20 ^e^	-	-
	DCM	9.50 ± 0.10 ^de^	20.00 ± 0.10 ^c^	-	-
	MeOH	10.00 ± 0.10 ^cd^	13.00 ± 0.20 ^g^	12.00 ± 0.10 ^b^	13.00 ± 0.10 ^b^
S.556	n-hexane	-	-	-	-
	EtOAc	6.50 ± 0.40 ^f^	14.50 ± 0.30 ^f^	-	-
	DCM	11.00 ± 0.10 ^ab^	21.50 ± 0.40 ^b^	-	-
	MeOH	10.00 ± 0.10 ^cd^	23.00 ± 0.20 ^a^	11.00 ± 0.20 ^c^	10.00 ± 0.10 ^d^
S.593	n-hexane	-	-	-	-
	EtOAc	10.50 ± 0.10 ^bc^	17.50 ± 0.30 ^d^	-	-
	DCM	10.50 ± 0.30 ^bc^	11.50 ± 0.20 ^h^	-	-
	MeOH	9.00 ± 0.30 ^e^	17.00 ± 0.20 ^d^	10.00 ± 0.20 ^d^	11.00 ± 0.10 ^c^

Notes: Values are expressed as the mean ± SD (*n* = 3). For *B. subtilis* and *X. oryzae*, different lowercase superscript letters within each column indicate significant differences among isolate–extract combinations according to two-way ANOVA followed by Tukey’s multiple-comparison test (*p* < 0.05); values sharing at least one letter are not significantly different. For *F. oxysporum* and *P. asparagi*, statistical comparisons were performed among the active MeOH extracts using one-way ANOVA followed by Tukey’s multiple-comparison test. “-” indicates no detectable antimicrobial activity.

**Table 3:** MIC and MBC values (against *B. subtilis* and *X. oryzae*) of the crude extracts of the cultures from the selected isolates.

		*B. subtilis*			*X. oryzae*		
Crude Extracts		MIC (mg/mL)	MBC (mg/mL)	MBC/MIC	MIC (mg/mL)	MBC (mg/mL)	MBC/MIC
S.002	EtOAc	6.25	25	4	6.25	25	4
	DCM	12.5	50	4	12.5	25	2
	MeOH	25	100	4	25	100	4
S.374	EtOAc	6.25	25	4	50	100	2
	DCM	25	100	4	25	100	4
	MeOH	25	100	4	50	100	2
S.556	EtOAc	50	100	2	50	100	2
	DCM	25	100	4	50	100	2
	MeOH	50	100	2	50	100	2
S.593	EtOAc	100	100	1	12.5	50	4
	DCM	50	100	2	50	100	2
	MeOH	100	100	1	50	100	2

**Table 4:** The antimicrobial activities of the 4 fractions (F1–F4) obtained from the EtOAc extract of S.002.

Fraction	Diameter of the Inhibition Zone (mm)			
	*B. subtilis*	*X. oryzae*	*F. oxysporum*	*P. asparagi*
**F1**	16.5 ± 0.2 mm	-	-	-
**F2**	-	-	-	-
**F3**	-	-	-	-
**F4**	-	19 ± 0.3 mm	-	-

**Table 5:** Tentatively identified compounds in fraction 4 of the EtOAc extract from isolate S.002 grouped by chemical class and selected bioactivity-related annotations.

Category	Compounds (No.–Name)	Notes
**Cyclic dipeptides**	1, 2, 7, 15, 21, 23	Microbial secondary metabolites with antimicrobial, cytotoxic or quorum-sensing- related activities [26,27]
**Cyclic lipopeptides**	12–Surfactin C, 14–Surfactin B, 24–Surfactin A	Antimicrobial lipopeptides [28]
**Cyclic lipopeptides** **(iturin family)**	22–Bacillomycin LC2	Antifungal lipopeptides [29]
**Lipoamino acids**	4, 5, 6, 9, 10, 11, 13, 16, 18	Membrane lipid and signaling [30]
**Oxidized lipoamino** **acids**	17, 20	Oxidized amphiphilic lipid metabolites [31]
**Alkaloids**	3–Norharmane, 8–Quinoline-4- carboxylic acid	Bioactive heteroaromatic metabolites [32,33]
**Amino acid derivative**	19–Tryptophan	Antibiofilm and growth inhibition [34]

## Data Availability

All essential data in this study are included in the article/Supplementary Materials. Further inquiries (regarding the raw LC–HRMS data, processed feature tables and GNPS workflows/task IDs) can be directed to the corresponding author and the respective data will be provided upon request.

## Supplementary Materials

The following supporting information can be downloaded at https://www.mdpi.com/article/10.3390/microorganisms14091994/s1: Figure S1: The agar diffusion assays showing the activities of the ethylacetate extracts of several selected macroalgae-associated isolates against the test microorganisms; Figure S2: Colony morphology and Gram-staining characteristics of the 15 selected seaweed-associated bacterial isolates; Figure S3: The agar diffusion assays showing the antagonistic activities against *B. subtilis* (left) and *X. oryzae* (right) of the fractions obtained from the ethylacetate extract of the S.002 culture; Table S1: Sampling information and geographic coordinates of macroalgal samples collected from the Cat Ba Archipelago, Hai Phong, Vietnam; Table S2: The antimicrobial activity of the selected isolates expressed as inhibition zone diameters against the test microorganisms; Table S3: 16S rRNA gene sequences of the selected bacterial isolates in this study and their corresponding GenBank accession numbers; Table S4: *rpoB* gene sequences of the selected *Bacillus* isolates in this study and their corresponding GenBank accession numbers; Table S5: LC–MS/MS characterization and tentative identification of compounds detected (in fraction F4) in the ethyl acetate extract of *Bacillus velezensis* S.002.
