# Supplementary material for: A Novel Discovery of Microorganisms Associated with Certain Seaweed Species from the Cat Ba Archipelago Area, Vietnam: Isolation, Identification and Exploration of Their Antimicrobial Activities
Source: Microorganisms. 2026 Sep 9;14(9):1994. doi: 10.3390/microorganisms14091994 (PMC13609368; doi:10.3390/microorganisms14091994)
Supplement: Supplementary file 1 [file microorganisms-14-01994-s001.zip › microorganisms-4492931-supplementary.pdf]

Supplementary Material

A novel discovery of microorganisms associated with certain seaweed species from the Cat Ba Archipelago area, Vietnam: isolation, identification and exploration of their antimicrobial activities

By Thanh Tien Tran et al.

Table S1. Sampling information and geographic coordinates of macroalgal samples collected from Cat Ba Archipelago, Hai Phong, Vietnam.

Species	Coordinates	Sampling location
Ulva flexuosa	20.801026° N<br>106.948944° E	Phu Long Dike, Cat Ba, Hai Phong
Valonia fastigiata	20.798333° N<br>107.065472° E	Inner inlet of Tung Gau Bay, Cat Ba, Hai Phong
Caulerpa racemosa	20.774556° N<br>107.073694° E	Hai Quan Inlet, Cat Ba, Hai Phong
Chaetomorpha linum	20.774556° N<br>107.073694° E	Hai Quan Inlet, Cat Ba, Hai Phong
Cladophora aokii	20.860444° N<br>106.986028° E	Gia Luan, Cat Ba, Hai Phong
Gelidium crinale	20.9850° N<br>107.0301° E	Gia Luan Ferry, Cat Ba, Hai Phong
Gracilaria tenuistipitata	20.801026° N<br>106.948944° E	Phu Long Dike, Cat Ba, Hai Phong
Dermonema pulvinatum	20.744776° N<br>107.05956° E	Tung Cu, Cat Ba, Hai Phong
Centroceras clavulatum	20.786083° N<br>107.075333° E	Tung Gau Station, Cat Ba, Hai Phong

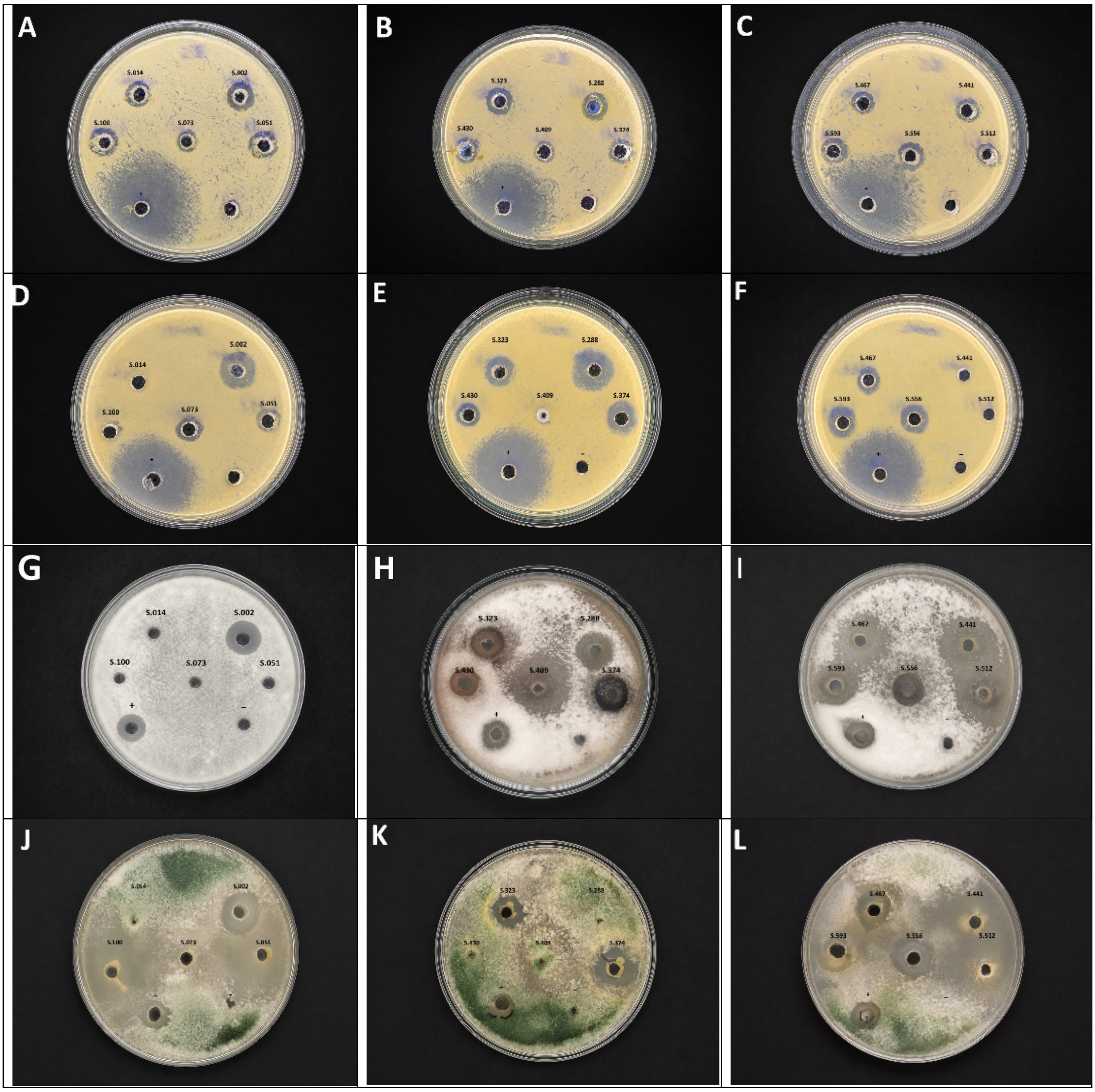

Figure S1. The agar diffusion assays showing the activities of the ethylacetate extracts of several selected macroalgae-associated isolates against the test microorganisms. Notes: (A–C) against *B. subtilis*; (D–F) against *X. oryzae*; (G-I) against *F. oxysporum*; (J-L) against *P. asparagi.* Erythromycin (10 mg/mL) was used as the positive control for the antibacterial assays, whereas salicylic acid (10 mg/mL) was used as the positive control for the antifungal assays. (-): negative control (the sterile medium only).

Table S2. The antimicrobial activity of the selected isolates expressed as inhibition-zone diameters against the test microorganisms. Notes: Values are expressed as mean ± SD. Within each column, different lowercase superscript letters indicate significant differences among isolates according to one-way ANOVA followed by Tukey's multiple-comparison test ($p < 0.05$); values sharing the same letter are not significantly different. A value of 5.00 mm corresponds to the well diameter and indicates no detectable inhibition beyond the well.

Isolate	*B. subtilis*	*X.oryzae*	*F.oxysporum*	*P. asparagi*
**S.002**	12.27 ± 0.25^{b}	14.17 ± 0.15^{b}	18.20 ± 0.20^{a}	17.77 ± 0.21^{c}
**S.014**	10.20 ± 0.20^{d}	5.00 ± 0.00^{i}	5.00 ± 0.00^{h}	5.00 ± 0.00^{g}
**S.051**	12.80 ± 0.20^{a}	8.23 ± 0.21^{h}	5.00 ± 0.00^{h}	5.00 ± 0.00^{g}
**S.073**	8.00 ± 0.10^{e}	11.17 ± 0.15^{e}	5.00 ± 0.00^{h}	5.00 ± 0.00^{g}
**S.100**	12.97 ± 0.15^{a}	5.00 ± 0.00^{i}	5.00 ± 0.00^{h}	5.00 ± 0.00^{g}
**S.288**	11.97 ± 0.15^{b}	15.13 ± 0.15^{a}	13.70 ± 0.26^{c}	5.00 ± 0.00^{g}
**S.323**	11.03 ± 0.15^{c}	12.20 ± 0.20^{d}	12.17 ± 0.15^{d}	16.27 ± 0.25^{d}
**S.374**	12.17 ± 0.15^{b}	12.13 ± 0.15^{d}	14.13 ± 0.15^{b}	18.30 ± 0.26^{b}
**S.409**	8.20 ± 0.20^{e}	5.00 ± 0.00^{i}	11.83 ± 0.15^{d}	5.00 ± 0.00^{g}
**S.430**	10.93 ± 0.12^{c}	9.17 ± 0.15^{g}	10.10 ± 0.10^{f}	5.00 ± 0.00^{g}
**S.441**	10.13 ± 0.15^{d}	5.00 ± 0.00^{i}	5.00 ± 0.00^{h}	5.00 ± 0.00^{g}
**S.467**	8.17 ± 0.15^{e}	10.23 ± 0.21^{f}	8.10 ± 0.10^{g}	13.17 ± 0.15^{f}
**S.512**	10.13 ± 0.15^{d}	5.00 ± 0.00^{i}	8.20 ± 0.20^{g}	5.00 ± 0.00^{g}
**S.556**	12.10 ± 0.10^{b}	12.17 ± 0.15^{d}	11.23 ± 0.21^{e}	19.10 ± 0.10^{a}
**S.593**	12.10 ± 0.17^{b}	13.13 ± 0.15^{c}	12.17 ± 0.15^{d}	14.23 ± 0.21^{e}

Isolation code	Colony morphology	Gram staining with *B. subtilis* as a control	Gram staining with *E. coli* as a control
S.002	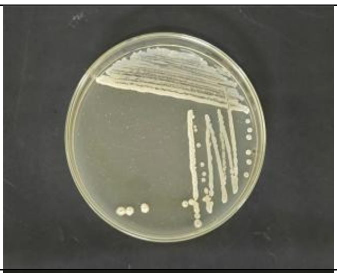	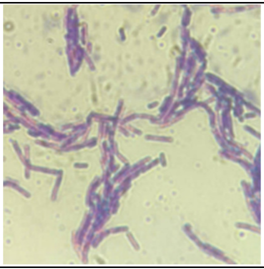	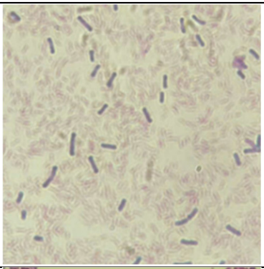
S.014	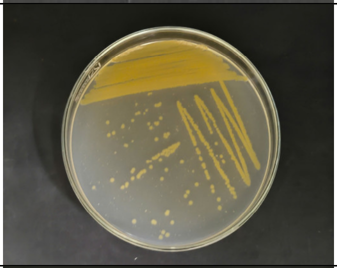	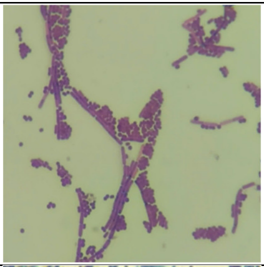	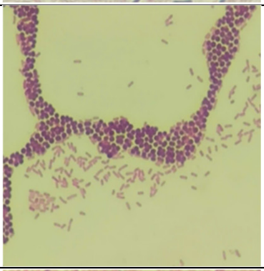
S.051	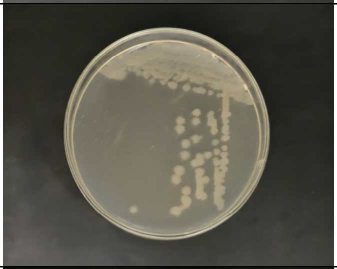	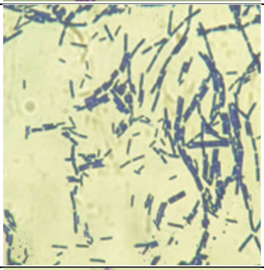	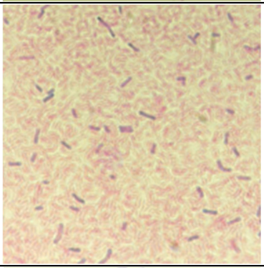
S.073	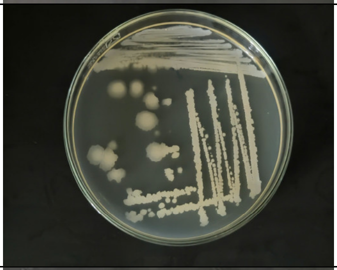	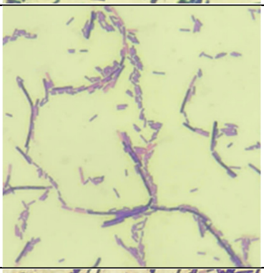	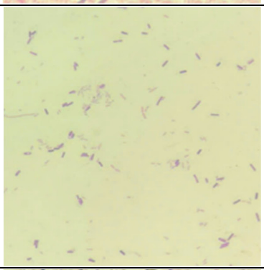
S.100	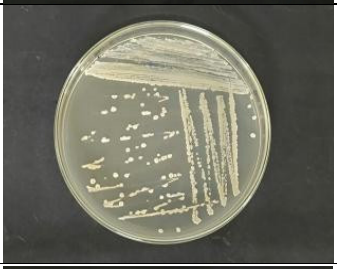	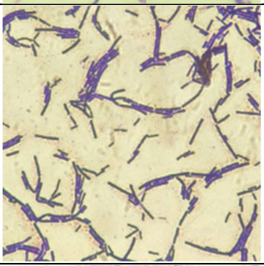	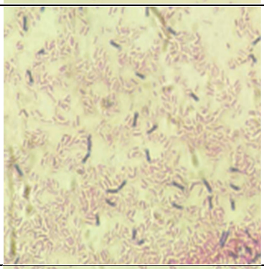
S.288	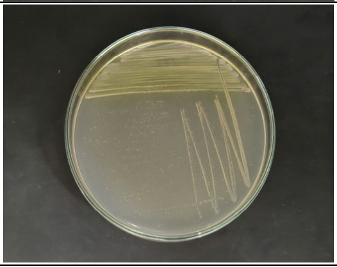	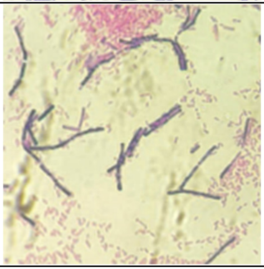	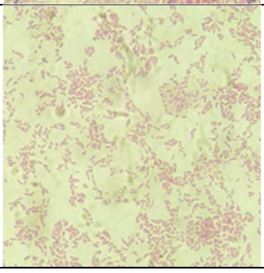

S.323	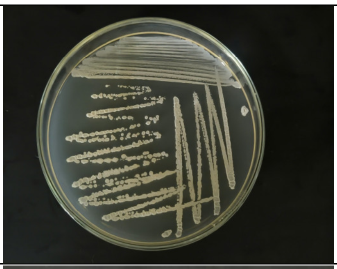	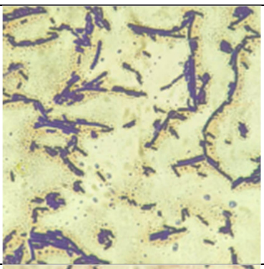	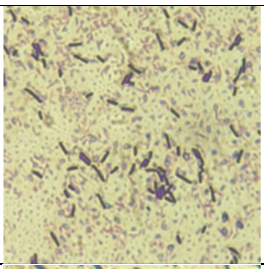
S.374	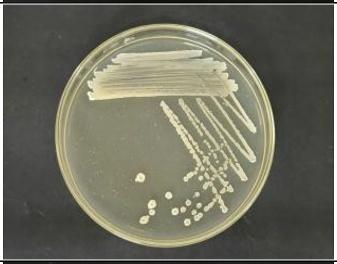	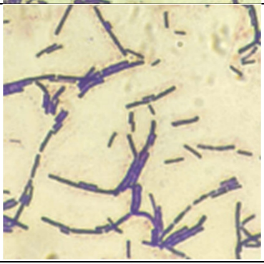	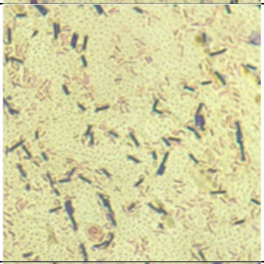
S.409	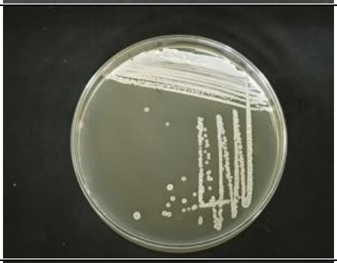	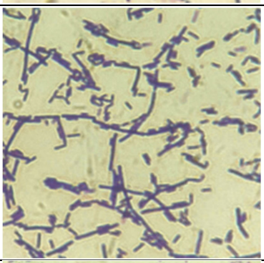	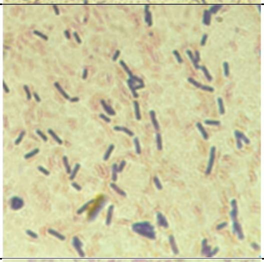
S.430	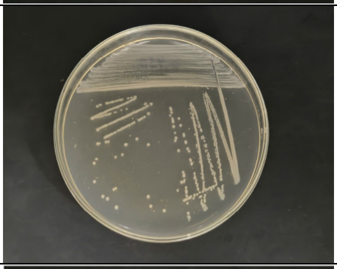	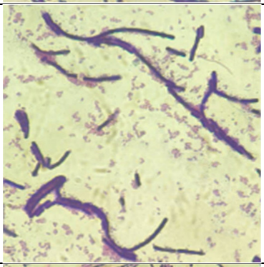	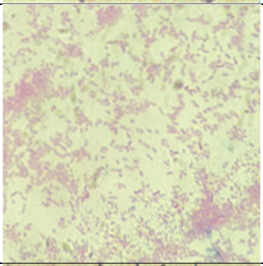
S.441	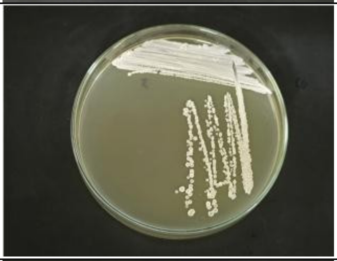	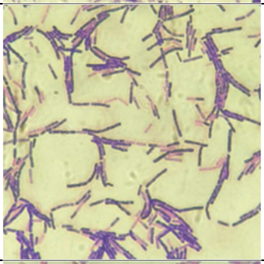	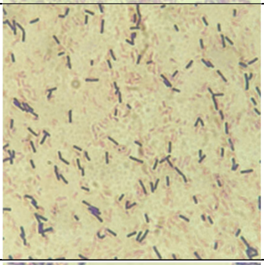
S.467	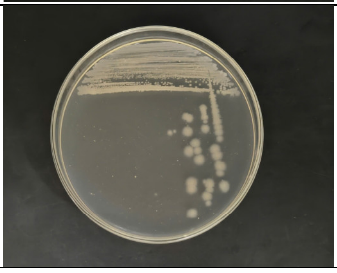	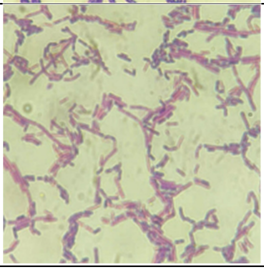	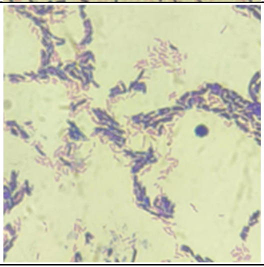

S.512	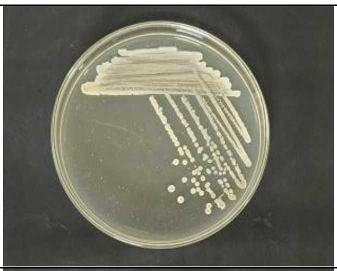	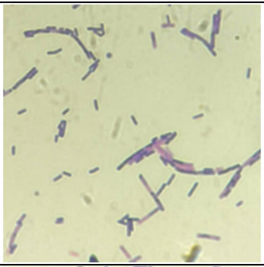	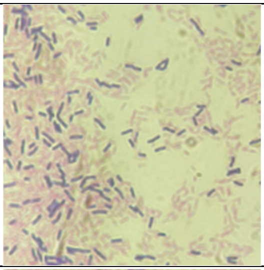
S.556	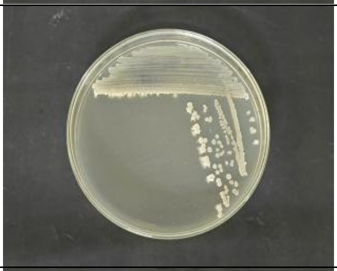	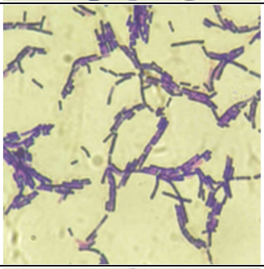	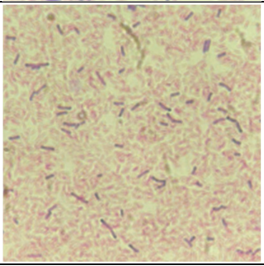
S.593	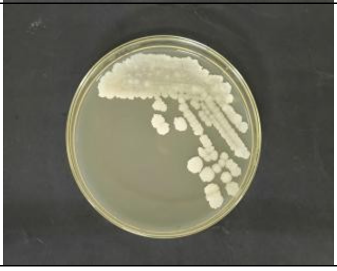	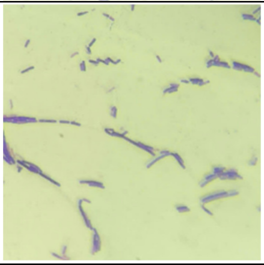	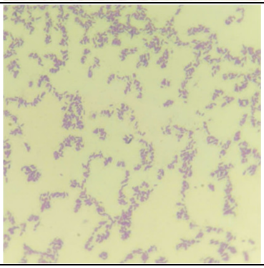

Figure S2. Colony morphology and Gram-staining characteristics of the 15 selected seaweed-associated bacterial isolates.

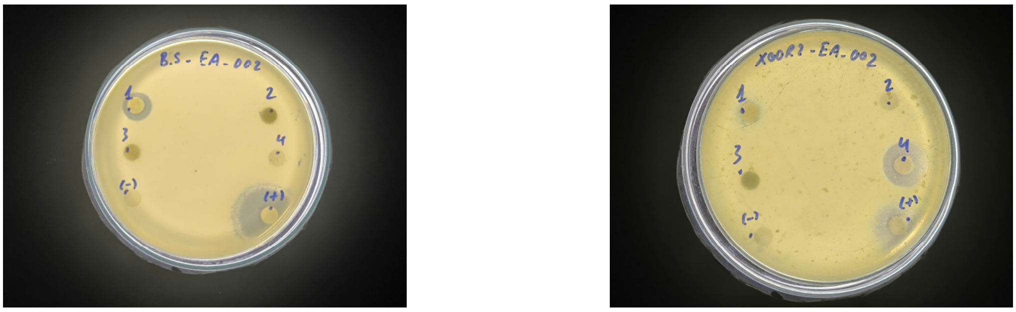

Figure S3. The agar diffusion assays showing the antagonistic activities against *B. subtilis* (left) and *X. oryzae* (right) of the fractions obtained from the ethylacetate extract of the S.002 culture. Notes: the numbers indicate the respective fractions F1-F4, (+): positive control (Erythomycin 10 mg/mL), (-): negative control (the sterile medium only).

Table S3. 16S rRNA gene sequences of the selected bacterial isolates in this study and their corresponding GenBank accession numbers

Isolate code	NCBI GenBank accession number	16S rRNA sequence (5’ – 3’)
S.002	PZ675836	AGATGGACCCGCGGCGCATTAGCTAGTTGGTGAGGTAACGGCTCACCAAGGCGACGATGCGTAGCCGACCTGAGAGGGTGATCGGCCACACT<br>GGGACTGAGACACGGCCCAGACTCCTACGGGAGGCAGCAGTAGGGAATCTTCCGCAATGGACGAAAGTCTGACGGAGCAACGCCGCGTGAGT<br>GATGAAGGTTTTCGGATCGTAAAGCTCTGTTGTTAGGGAAGAACAAGTGCCGTTCAAATAGGGCGGCACCTTGACGGTACCTAACCAGAAAGC<br>CACGGCTAACTACGTGCCAGCAGCCGCGGTAATACGTAGGTGGCAAGCGTTGTCCGGAATTATTGGGCGTAAAGGGCTCGCAGGCGGTTTCTT<br>AAGTCTGATGTGAAAGCCCCCGGCTCAACCGGGGAGGGTCATTGGAAACTGGGGAACTTGAGTGCAGAAGAGGAGAGTGGAATTCCACGTGT<br>AGCGGTGAAATGCGTAGAGATGTGGAGGAACACCAGTGGCGAAGGCGACTCTCTGGTCTGTAACTGACGCTGAGGAGCGAAAGCGTGGGGAG<br>CGAACAGGATTAGATACCCTGGTAGTCCACGCCGTAAACGATGAGTGCTAAGTGTTAGGGGGTTTCCGCCCCTTAGTGCTGCAGCTAACGCAT<br>TAAGCACTCCGCCTGGGGAGTACGGTCGCAAGACTGAAACTCAAAGGAATTGACGGGGGCCCGCACAAGCGGTGGAGCATGTGGTTTAATTC<br>GAAGCAACGCGAAGAACCTTACCAGGTCTTGACATCCTCTGACAATCCTAGAGATAGGACGTCCCCTTCGGGGGCAGAGTGACAGGTGGTGC<br>ATGGTTGTCGTCAGCTCGTGTCGTGAGATGTTGGGTTAAGTCCCGCAACGAGCGCAACCCTTGATCTTAGTTGCCAGCATTCAGTTGGGCACTC<br>TAAGGTGACTGCCGGTGACAAACCGGAGGAAGGTGGGGATGACGTCAAATCATCATGCCCCTTATGACCTGGGCTACACACGTGCTACAATG<br>GACAGAACAAAGGGCAGCGAAACCGCGAGGTTAAGCCAATCCCACAAATCTGTTCTCAGTTCGGATCGCAGTCTGCAACTCGACTGCGTGAA<br>GCTGGAATC
S.014	PZ702527	GTCACCTTGAGATGGCCTTGCGGTGCATTAGCTAGTTGGTGGGGTAACGGCCCACCAAGGCGACGATGCATAGCCGACCTGAGAGGGTGATCG<br>GCCACACTGGGACTGAGACACGGCCCAGACTCCTACGGGAGGCAGCAGTAGGGAATCTTCCACAATGGACGAAAGTCTGATGGAGCAACGCC<br>GCGTGAGTGATGAAGGTTTTCGGATCGTAAAACTCTGTTGTAAGGGAAGAACACGTACGAGAGGGAATGCTCGTACCTTGACGGTACCTTACG<br>AGAAAGCCACGGCTAACTACGTGCCAGCAGCCGCGGTAATACGTAGGTGGCAAGCGTTGTCCGGAATTATTGGGCGTAAAGCGCGCGCAGGC<br>GGCCTTTTAAGTCTGATGTGAAAGCCCCCGGCTCAACCGGGGAGGGCCATTGGAAACTGGAAGGCTTGAGTACAGAAGAGAAGAGTGGAATT<br>CCACGTGTAGCGGTGAAATGCGTAGAGATGTGGAGGAACACCAGTGGCGAAGGCGACTCTTTGGTCTGTAACTGACGCTGAGGCGCGAAAGC<br>GTGGGGAGCAAACAGGATTAGATACCCTGGTAGTCCACGCCGTAAACGATGAGTGCTAGTGTGTTGGGGGGTTTCCGCCCCTCAGTGCTGAAG<br>CTAACGCATTAAGCACTCCGCCTGGGGAGTACGGCCGCAAGGCTGAAACTCAAAGGAATTGACGGGGACCCGCACAAGCGGTGGAGCATGTG

		GTTTAATTCGAAGCAACGCGAAGAACCTTACCAACTCTTGACATCCCATTGACCGCTTGAGAGATCAAGTTTTCCCTTCGGGGACAATGGTGAC<br>AGGTGGTGCATGGTTGTCGTCAGCTCGTGTCGTGAGATGTTGGGTTAAGTCCCGCAACGAGCGCAACCCCTATCCTTAGTTGCCAGCATTCAGT<br>TGGGCACTCTAGGGAGACTGCCGGTGACAAACCGGAGGAAGGTGGGGATGACGTCAAATCATCATGCCCCTTATGAGTTGGGCTACACACGT<br>GCTACAATGGACGGTACAAAGGGCAGCGAGACCGCGAGGTGGAGCCAATCCCATAAAGCCGTTCCCAGTTCGGATTGCAGGCTGCAACTCGC<br>CTGCATGAAGTCGGAATCGCTAGTAATCGCAGGTCAGCAT
S.051	PZ702530	CGTAGCCGACCTGAGAGGGTGATCGGCCACACTGGGACTGAGACACGGCCCAGACTCCTACGGGAGGCAGCAGTAGGGAATCTTCCGCAATG<br>GACGAAAGTCTGACGGAGCAACGCCGCGTGAGTGATGAAGGTTTTCGGATCGTAAAGCTCTGTTGTTAGGGAAGAACAAGTACCGTTCGAAT<br>AGGGCGGTACCTTGACGGTACCTAACCAGAAAGCCACGGCTAACTACGTGCCAGCAGCCGCGGTAATACGTAGGTGGCAAGCGTTGTCCGGA<br>ATTATTGGGCGTAAAGGGCTCGCAGGCGGTTTCTTAAGTCTGATGTGAAAGCCCCCGGCTCAACCGGGGAGGGTCATTGGAAACTGGGGAACT<br>TGAGTGCAGAAGAGGAGAGTGGAATTCCACGTGTAGCGGTGAAATGCGTAGAGATGTGGAGGAACACCAGTGGCGAAGGCGACTCTCTGGTC<br>TGTAACTGACGCTGAGGAGCGAAAGCGTGGGGAGCGAACAGGATTAGATACCCTGGTAGTCCACGCCGTAAACGATGAGTGCTAAGTGTTAG<br>GGGGTTTCCGCCCCTTAGTGCTGCAGCTAACGCATTAAGCACTCCGCCTGGGGAGTACGGTCGCAAGACTGAAACTCAAAGGAATTGACGGGG<br>GCCCGCACAAGCGGTGGAGCATGTGGTTTAATTCGAAGCAACGCGAAGAACCTTACCAGGTCTTGACATCCTCTGACAATCCTAGAGATAGGA<br>CGTCCCCTTCGGGGGCAGAGTGACAGGTGGTGCATGGTTGTCGTCAGCTCGTGTCGTGAGATGTTGGGTTAAGTCCCGCAACGAGCGCAACCC<br>TTGATCTTAGTTGCCAGCATTCAGTTGGGCACTCTAAGGTGACTGCCGGTGACAAACCGGAGGAAGGTGGGGATGACGTCAAATCATCATGCC<br>CCTTATGACCTGGGCTACACACGTGCTACAATGGACAGAACAAAGGGCAGCGAAACCGCGAGGTTAAGCCAATCCCACAAATCTGTTCTCAGT<br>TCGGATCGCAGTCTGCAACTCGACTGCGTGAAGCTGGAATCGCTAGTAATCGCGGATCAGCA
S.073	PZ702531	CTACCTGTAAGACTGGGATAACTCCGGGAAACCGGGGCTAATACCGGATGAAACAAAGCGTCGCATGACGCAATGTTAAAAGGCGGCATATG<br>CTGTCACTTACAGATGGGCCCGCGGCGCATTAGCTAGTTGGTGAGGTAAAGGCTCACCAAGGCAACGATGCGTAGCCGACCTGAGAGGGTGA<br>TCGGCCACACTGGGACTGAGACACGGCCCAGACTCCTACGGGAGGCAGCAGTAGGGAATCTTCCGCAATGGACGAAAGTCTGACGGAGCAAC<br>GCCGCGTGAGTGATGAAGGTTTTCGGATCGTAAAACTCTGTTGTTAGGGAAGAACAAGTGCCATTCGAATAGGTTGGCACCTTGACGGTACCT<br>AACCAGAAAGCCCCGGCTAACTACGTGCCAGCAGCCGCGGTAATACGTAGGGGGCAAGCGTTGTCCGGAATTATTGGGCGTAAAGCGCGCGC<br>AGGCGGTCCTTTAAGTCTGATGTGAAAGCCCACGGCTTAACCGTGGAGGGCCATTGGAAACTGGAGGACTTGAGTACAGAAGAGGAGAGTGG<br>AATTCCACGTGTAGCGGTGAAATGCGTAGAGATGTGGAGGAACACCAGTGGCGAAGGCGACTCTCTGGTCTGTAACTGACGCTGAGGTGCGA<br>AAGCGTGGGTAGCGAACAGGATTAGATACCCTGGTAGTCCACGCCGTAAACGATGAGTGCTAGGTGTTAAGGGGGGTTTCCGCCCCTTAGTGC<br>TGAAGTTAACGCATTAAGCACTCCGCCTGGGGAGTACGGCCGCAAGGCTGAAACTCAAAAGAATTGACGGGGACCCGCACAAGCGGTGGAGC

		ATGTGGTTTAATTCGAAGCAACGCGAAGAACCTTACCAGGTCTTGACATCCTCTGACACCCCTAGAGATAGGGCATTCCCTTCGGGGACAGAGTGACAGGTGGTGCATGGTTGTCGTCAGCTCGTGTCGTGAGATGTTGGGTTAAGTCCCGCAACGAGCGCAACCCTTGATCTTAGTTGCCAGCATTTAGTTGGGCACTCTAAGGTGACTGCCGGTGACAAACCGGAGGAAGGTGGGGATGACGTCAAATCATCATGCCCCTTATGACCTGGGCTACACACGTGCTACAATGGATGGAACAAAGGGCAGCGAAGCCGCGAGGCCAAGCAAATCCCATAAAACCATTCTCAGTTCGGATTGCAGGCTGCAACTCGCCTGCATGAAGCCGGAATCGCTAGTAATCGCGGATCA
S.100	PZ675722	GGAAACCGGGGCTAATACCGGATGCTTGTTTGAACCGCATGGTTCAAACATAAAAGGTGGCTTCGGCTACCACTTACAGATGGACCCGCGGCGCATTAGCTAGTTGGTGAGGTAATGGCTCACCAAGGCAACGATGCGTAGCCGACCTGAGAGGGTGATCGGCCACACTGGGACTGAGACACGGCCCAGACTCCTACGGGAGGCAGCAGTAGGGAATCTTCCGCAATGGACGAAAGTCTGACGGAGCAACGCCGCGTGAGTGATGAAGGTTTTCGGATCGTAAAGCTCTGTTGTTAGGGAAGAACAAGTACCGTTCGAATAGGGCGGTACCTTGACGGTACCTAACCAGAAAGCCACGGCTAACTACGTGCCAGCAGCCGCGGTAATACGTAGGTGGCAAGCGTTGTCCGGAATTATTGGGCGTAAAGGGCTCGCAGGCGGTTTCTTAAGTCTGATGTGAAAGCCCCCGGCTCAACCGGGGAGGGTCATTGGAAACTGGGGAACTTGAGTGCAGAAGAGGAGAGTGGAATTCCACGTGTAGCGGTGAAATGCGTAGAGATGTGGAGGAACACCAGTGGCGAAGGCGACTCTCTGGTCTGTAACTGACGCTGAGGAGCGAAAGCGTGGGGAGCGAACAGGATTAGATACCCTGGTAGTCCACGCCGTAAACGATGAGTGCTAAGTGTTAGGGGGTTTCCGCCCCTTAGTGCTGCAGCTAACGCATTAAGCACTCCGCCTGGGGAGTACGGTCGCAAGACTGAAACTCAAAGGAATTGACGGGGGCCCGCACAAGCGGTGGAGCATGTGGTTTAATTCGAAGCAACGCGAAGAACCTTACCAGGTCTTGACATCCTCTGACAATCCTAGAGATAGGACGTCCCCTTCGGGGGCAGAGTGACAGGTGGTGCATGGTTGTCGTCAGCTCGTGTCGTGAGATGTTGGGTTAAGTCCCGCAACGAGCGCAACCCTTGATCTTAGTTGCCAGCATTCAGTTGGGCACTCTAAGGTGACTGCCGGTGACAAACCGGAGGAAGGTGGGGATGACGTCAAATCATCATGCCCCTTATGACCTGGGCTACACACGTGCTACAATGGACAGAACAAAGGGCAGCGAAACCGCGAGGTTAAGCCAATCCCACAAATCTGTTCTCAGTTCGGATCGCAGTCTGCAACTCGACTGCGTGAAGCTGGA
S.288	PZ702538	GTGCCCAGTAGCGGGGGATAACTACTCGAAAGAGTGGCTAATACCGCATACGCCCTACGGGGGAAAGGGGGGGATTCTTCGGAACCTCTCACTATTGGAGCGGCCGATATCGGATTAGCTAGTTGGTGGGGTAAAGGCTCACCAAGGCAACGATCCGTAGCTGGTTTGAGAGGACGACCAGCCACACTGGGACTGAGACACGGCCCAGACTCCTACGGGAGGCAGCAGTGGGGAATTTTGGACAATGGGGGAAACCCTGATCCAGCCATCCCGCGTGTATGATGAAGGCCTTCGGGTTGTAAAGTACTTTTGGCAGAGAAGAAAAGGTATCTCCTAATACGAGATACTGCTGACGGTATCTGCAGAATAAGCACCGGCTAACTACGTGCCAGCAGCCGCGGTAATACGTAGGGTGCAAGCGTTAATCGGAATTACTGGGCGTAAAGCGTGTGTAGGCGGTTCGGAAAGAAAGATGTGAAATCCCAGGGCTCAACCTTGGAACTGCATTTTTAACTGCCGAGCTAGAGTATGTCAGAGGGGGGTAGAATTCCACGTGTAGCAGTGAAATGCGTAGATATGTGGAGGAATACCGATGGCGAAGGCAGCCCCCTGGGATAATACTGACGCTCAGACACGAAAGCGTGGGG

		AGCAAACAGGATTAGATACCCTGGTAGTCCACGCCCTAAACGATGTCAACTAGCTGTTGGGGCCGTTAGGCCTTAGTAGCGCAGCTAACGCGT<br>GAAGTTGACCGCCTGGGGAGTACGGTCGCAAGATTAAAACTCAAAGGAATTGACGGGGACCCGCACAAGCGGTGGATGATGTGGATTAATTC<br>GATGCAACGCGAAAAACCTTACCTACCCTTGACATGTCTGGAATGCCGAAGAGATTTGGCAGTGCTCGCAAGAGAACCGGAACACAGGTGCT<br>GCATGGCTGTCGTCAGCTCGTGTCGTGAGATGTTGGGTTAAGTCCCGCAACGAGCGCAACCCTTGTCATTAGTTGCTACGCAAGAGCACTCTAA<br>TGAGACTGCCGGTGACAAACCGGAGGAAGGTGGGGATGACGTCAAGTCCTCATGGCCCTTATGGGTAGGGCTTCACACGTCATACAATGGTCG<br>GGACAGAGGGTCGCCAACCCGCGAGGGGGAGCCAATCTCAGAAACCCGATCGTAGTCCGGATCGCAGTCTGCAACTCGACTGCGTGAAGTCG<br>GAATCGCTA
S.323	PZ702539	CCTGAGAGGGTGATCGGCCACACTGGGACTGAGACACGGCCCAGACTCCTACGGGAGGCAGCAGTAGGGAATCTTCCGCAATGGACGAAAGT<br>CTGACGGAGCAACGCCGCGTGAGTGATGAAGGTTTTCGGATCGTAAAGCTCTGTTGTTAGGGAAGAACAAGTGCCGTTCAAATAGGGCGGCA<br>CCTTGACGGTACCTAACCAGAAAGCCACGGCTAACTACGTGCCAGCAGCCGCGGTAATACGTAGGTGGCAAGCGTTGTCCGGAATTATTGGGC<br>GTAAAGGGCTCGCAGGCGGTTTCTTAAGTCTGATGTGAAAGCCCCCGGCTCAACCGGGGAGGGTCATTGGAAACTGGGGAACTTGAGTGCAG<br>AAGAGGAGAGTGGAATTCCACGTGTAGCGGTGAAATGCGTAGAGATGTGGAGGAACACCAGTGGCGAAGGCGACTCTCTGGTCTGTAACTGA<br>CGCTGAGGAGCGAAAGCGTGGGGAGCGAACAGGATTAGATACCCTGGTAGTCCACGCCGTAAACGATGAGTGCTAAGTGTTAGGGGGTTTCC<br>GCCCCTTAGTGCTGCAGCTAACGCATTAAGCACTCCGCCTGGGGAGTACGGTCGCAAGACTGAAACTCAAAGGAATTGACGGGGGCCCGCAC<br>AAGCGGTGGAGCATGTGGTTTAATTCGAAGCAACGCGAAGAACCTTACCAGGTCTTGACATCCTCTGACAATCCTAGAGATAGGACGTCCCCT<br>TCGGGGGCAGAGTGACAGGTGGTGCATGGTTGTCGTCAGCTCGTGTCGTGAGATGTTGGGTTAAGTCCCGCAACGAGCGCAACCCTTGATCTT<br>AGTTGCCAGCATTCAGTTGGGCACTCTAAGGTGACTGCCGGTGACAAACCGGAGGAAGGTGGGGATGACGTCAAATCATCATGCCCCTTATGA<br>CCTGGGCT
S.374	PZ702540	TAGCGGCGGACGGGTGAGTAACACGTGGGTAACCTGCCTGTAAGACTGGGATAACTCCGGGAAACCGGGGCTAATACCGGATGGTTGTCTGA<br>ACCGCATGGTTCAGACATAAAAGGTGGCTTCGGCTACCACTTACAGATGGACCCGCGGCGCATTAGCTAGTTGGTGAGGTAACGGCTCACCAA<br>GGCGACGATGCGTAGCCGACCTGAGAGGGTGATCGGCCACACTGGGACTGAGACACGGCCCAGACTCCTACGGGAGGCAGCAGTAGGGAATC<br>TTCCGCAATGGACGAAAGTCTGACGGAGCAACGCCGCGTGAGTGATGAAGGTTTTCGGATCGTAAAGCTCTGTTGTTAGGGAAGAACAAGTGC<br>CGTTCAAATAGGGCGGCACCTTGACGGTACCTAACCAGAAAGCCACGGCTAACTACGTGCCAGCAGCCGCGGTAATACGTAGGTGGCAAGCG<br>TTGTCCGGAATTATTGGGCGTAAAGGGCTCGCAGGCGGTTTCTTAAGTCTGATGTGAAAGCCCCCGGCTCAACCGGGGAGGGTCATTGGAAAC<br>TGGGGAACTTGAGTGCAGAAGAGGAGAGTGGAATTCCACGTGTAGCGGTGAAATGCGTAGAGATGTGGAGGAACACCAGTGGCGAAGGCGA<br>CTCTCTGGTCTGTAACTGACGCTGAGGAGCGAAAGCGTGGGGAGCGAACAGGATTAGATACCCTGGTAGTCCACGCCGTAAACGATGAGTGCT

		AAGTGTTAGGGGGTTTCCGCCCCTTAGTGCTGCAGCTAACGCATTAAGCACTCCGCCTGGGGAGTACGGTCGCAAGACTGAAACTCAAAGGAATTGACGGGGGCCCGCACAAGCGGTGGAGCATGTGGTTTAATTCGAAGCAACGCGAAGAACCTTACCAGGTCTTGACATCCTCTGACAATCCTAGAGATAGGACGTCCCCTTCGGGGGCAGAGTGACAGGTGGTGCATGGTTGTCGTCAGCTCGTGTCGTGAGATGTTGGGTTAAGTCCCGCAACGAGCGCAACCCTTGATCTTAGTTGCCAGCATTCAGTTGGGCACTCTAAGGTGACTGCCGGTGACAAACCGAGAGGAAGGTGGGGATGACGTCAAATCATCATGCCCCTTATGACCTGGGCTACACACGTGCTACAATGGACAGAACAAAGGGCAGCGAAACCGCGAGGTTAAGCCAATCCCACAAATCTGTTCTCAGTTCGGATCGCAGTCTGCAACTCGACTGCGTGAAGCTGGAATCGCTAGTAATCGCGGATCAGCATGCCGCGGTGAATACG
S.409	PZ702542	CTAGTTGGTGAGGTAACGGCTCACCAAGG-AACGAT-CGTA-CCGACCTGAGAGGGT-ATCG-CCAC-CTGGGACTGA-ACACGGCCCAGACTCCT-CGGGAG-CAGCAGTAGG-AATCTTCCGCAATG-ACGAAAGTCTGACGGAGCAACGCCGCGTGAGTGATGAAGGTTTTCGGATCGTAA-GCTCTGTTGTTAGG-AAGAACAAGTACCGTTCGAATAGGGCGGTACCTTGACGGTACCTAACCAGAAAGCCACGGCTAACTACGTGCCAGCAGCCGCGGTAATACGTAGGTGGCAAGCGTTGTCCGGAATTATTGGGCGTAAAGGGCTCGCAGGCGGTTTCTTAAGTCTGATGTGAAAGCCCCCGGCTCAACCGGGGAGGGTCATTGGAAACTGGGGAACTTGAGTGCAGAAGAGGAGAGTGGAATTCCACGTGTAGCGGTGAAATGCGTAGAGATGTGGAGGAACACCAGTGGCGAAGGCGACTCTCTGGTCTGTAACTGACGCTGAGGAGCGAAAGCGTGGGGAGCGAACAGGATTAGATACCCTGGTAGTCCACGCCGTAAACGATGAGTGCTAAGTGTTAGGGGGTTTCCGCCCCTTAGTGCTGCAGCTAACGCATTAAGCACTCCGCCTGGGGAGTACGGTCGCAAGACTGAAACTCAAAGGAATTGACGGGGGCCCGCACAAGCGGTGGAGCATGTGGTTTAATTCGAAGCAACGCGAAGAACCTTACCAGGTCTTGACATCCTCTGACAATCCTAGAGATAGGACGTCCCCTTCGGGGGCAGAGTGACAGGTGGTGCATGGTTGTCGTCAGCTCGTGTCGTGAGATGTTGGGTTAAGTCCCGCAACGAGCGCAACCCTTGATCTTAGTTGCCAGCATTCAGTTGGGCACTCTAAGGTGACTGCCGGTGACAAACCGGAGGAAGGTGGGGATGACGTCAAATCATCATGCCCCTTATGACCTGGGCTACACACGTGCTACAATGGACAGAACAAAGGGCAGCGAAACCGCGAGGTTAAGCCAATCCCACAAATCTGTTCTCAGTTCGGATCGCAGTCTGCA-ACTCGACT
S.430	PZ742062	GCAATGGGCGCAAGCCTGATGCAGCCATGCCGCGTGTGTGAAGAAGGCCTTCGGGTTGTAAAGCACTTTCAGCGAGNGAGGAAGGTGGTGAGCTTAATACGCTCATCAATTGACGTTACTCGCNAGAAGAAGCACCGGCTAACTCCGTGCCAGCAGCCGCGGTAATACGGAGGGTGCAAGCGTTAATCGGAATTACTGGGCGTAAAGCGCACGCAGGCGGTNTTGTTAAGTCAGATGTGAAATCCCCGGGCTCAACCTGGGAACTGCATTTGAAACTGGCAAGCTAGAGTCTCGTAGAGGGGGGTAGAATTCCAGGTGTAGCGGTGAAATGCGTAGAGATCTGGAGGAATACCGGTGGCGAAGGCGGCCCCCTGGACGAAGACNTGACGCTCAGGTGCGAAAGNNNCGTGGGGAGCAAACAGGATTAGATACCCTGGTAGTCCACGCTGTAAACGATGTCGATTTGGAGGTTGTGCCCTTGAGGCGTGGCTTCCGGAGCTAACGCGTTAAATCGACCGCCTGGGGAGTACGGCCGCAAGGTTAAAACNTCAAATG

		AATTGACGGGGGCCCGCACAAGCGGTGGAGCATGTGGTTTAATTCGATGCANACGCGAAGAACCTTACNCTACTCTTGACATCCAGAGAACTT<br>TCCAGAGATGGATTGGTGCCTTCGGGAACTCTGAGACAGGTGCTGCATGGCTGTCGTCAGCTCGTGTTGTGAAATGTTGGGTTAAGTCCCGCA<br>ACGAGCGCAACCCTTATCCTTTGTTGCCAGCGGTTCGGCCGGGAACTCAAAGGAGACTGCCAGTGATAAACTGGAGGAAGGTGGGGATGACG<br>TCAAGTCATCATGGCCCTTNNACGAGTAGGGCTACACACGTGCTACAATGGCGTATACAAAGAGAAGCGACCTCGCGAGAGCAANGCGGACC<br>TCATAAAGTACGTCGTAGTCCGGATTGGAGTCTGCAACTCNNGACTCCATGAAGTCGGAATCGCTAGTAATCGTAGATCAGAATGCTACGNGT<br>GAATACGTTCCCGGGCCTTGTACACACCGCCCGTCACACCATGGGAGTGGGTTGCAAAAGAAGTAGGTAGCTTAACCTTCGGGAGGAGCGCGT<br>CTACGCGAACACTTGTTCCACGG
S.441	PZ702544	CGCATTAGCTAGTTGGTGAGGTAACG--TCACCAAGGC-ACGATG-GTAGCCGACCTGAGAGGGT-ATCGGCCAC-CTGGGACTGA-<br>ACACGGCCCAGACTCCT-CGGGAGGCAGCAGTAGG-AATCTTCCGCAATGGACGAAAGTCT-ACGGAGCAACGCCGCGTGAGT-<br>ATGAAGGTTTTCGGATCGTAAAGCTCTGTTGTTAGGGAAGAACAAGTGCCGTTCAAATAGGGCGGCACCTTGACGGTACCTAACCAGAAAGCC<br>ACGGCTAACTACGTGCCAGCAGCCGCGGTAATACGTAGGTGGCAAGCGTTGTCCGGAATTATTGGGCGTAAAGGGCTCGCAGGCGGTTTCTTA<br>AGTCTGATGTGAAAGCCCCCGGCTCAACCGGGGAGGGTCATTGGAAACTGGGGAACTTGAGTGCAGAAGAGGAGAGTGGAATTCCACGTGTA<br>GCGGTGAAATGCGTAGAGATGTGGAGGAACACCAGTGGCGAAGGCGACTCTCTGGTCTGTAACTGACGCTGAGGAGCGAAAGCGTGGGGAGC<br>GAACAGGATTAGATACCCTGGTAGTCCACGCCGTAAACGATGAGTGCTAAGTGTTAGGGGGTTTCCGCCCCTTAGTGCTGCAGCTAACGCATT<br>AAGCACTCCGCCTGGGGAGTACGGTCGCAAGACTGAAACTCAAAGGAATTGACGGGGGCCCGCACAAGCGGTGGAGCATGTGGTTTAATTCG<br>AAGCAACGCGAAGAACCTTACCAGGTCTTGACATCCTCTGACAATCCTAGAGATAGGACGTCCCCTTCGGGGGCAGAGTGACAGGTGGTGCAT<br>GGTTGTCGTCAGCTCGTGTCGTGAGATGTTGGGTTAAGTCCCGCAACGAGCGCAACCCTTGATCTTAGTTGCCAGCATTCAGTTGGGCACTCTA<br>AGGTGACTGCCGGTGACAAACCGGAGGAAGGTGGGGATGACGTCAAATCATCATGCCCCTTATGACCTGGGCTACACACGTGCTACAATGGG<br>CAGAACAAAGGGCAGCGAAACCGCGAGGTTAA
S.467	PZ702545	TTCAAATAGGGCGGCACCTTGACGGTACCTAACCAGAAAGCCACGGCTAACTACGTGCCAGCAGCCGCGGTAATACGTAGGTGGCAAGCGTT<br>GTCCGGAATTATTGGGCGTAAAGGGCTCGCAGGCGGTTTCTTAAGTCTGATGTGAAAGCCCCCGGCTCAACCGGGGAGGGTCATTGGAAACTG<br>GGGAACTTGAGTGCAGAAGAGGAGAGTGGAATTCCACGTGTAGCGGTGAAATGCGTAGAGATGTGGAGGAACACCAGTGGCGAAGGCGACT<br>CTCTGGTCTGTAACTGACGCTGAGGAGCGAAAGCGTGGGGAGCGAACAGGATTAGATACCCTGGTAGTCCACGCCGTAAACGATGAGTGCTA<br>AGTGTTAGGGGGTTTCCGCCCCTTAGTGCTGCAGCTAACGCATTAAGCACTCCGCCTGGGGAGTACGGTCGCAAGACTGAAACTCAAAGGAAT<br>TGACGGGGGCCCGCACAAGCGGTGGAGCATGTGGTTTAATTCGAAGCAACGCGAAGAACCTTACCAGGTCTTGACATCCTCTGACAATCCTAG<br>AGATAGGACGTCCCCTTCGGGGGCAGAGTGACAGGTGGTGCATGGTTGTCGTCAGCTCGTGTCGTGAGATGTTGGGTTAAGTCCCGCAA--

		CGAGCGCAACCCTTGATCTTAGTTGCCAGCATTCAGTTGGGCACTCTAAGGTGACTGCCGGTGACAAACCGGAGGAAGGTGGGGATGACGTCA<br>AATCATCATGCCCCTTATGACCTGGGCTACACACGTGCTACAATGGGCAGAACAAAGGGCAGCGAAACCGCGAGGTTAAGCCAATCCCACAA<br>ATCTGTTCTCAGTTCGGATCGCAGTCTGCAACTCGACTGCGTGAAGCTGGAATCGCTAGTAATCGCGGATCAGCATGCCGCGG
S.512	PZ702556	TGATGTTAGCGGCGGACGGGTGAGTAACACGTGGGTAACCTGCCTGTAAGACTGGGATAACTCCGGGAAACCGGGGCTAATACCGGATGGTT<br>GTTTGAACCGCATGGTTCAAACATAAAAGGTGGCTTCGGCTACCACTTACAGATGGACCCGCGGCGCATTAGCTAGTTGGTGAGGTAACGGCT<br>CACCAAGGCAACGATGCGTAGCCGACCTGAGAGGGTGATCGGCCACACTGGGACTGAGACACGGCCCAGACTCCTACGGGAGGCAGCAGTAG<br>GGAATCTTCCGCAATGGACGAAAGTCTGACGGAGCAACGCCGCGTGAGTGATGAAGGTTTTCGGATCGTAAAGCTCTGTTGTTAGGGAAGAAC<br>AAGTACCGTTCGAATAGGGCGGTACCTTGACGGTACCTAACCAGAAAGCCACGGCTAACTACGTGCCAGCAGCCGCGGTAATACGTAGGTGG<br>CAAGCGTTGTCCGGAATTATTGGGCGTAAAGGGCTCGCAGGCGGTTTCTTAAGTCTGATGTGAAAGCCCCCGGCTCAACCGGGGAGGGTCATT<br>GGAAACTGGGGAACTTGAGTGCAGAAGAGGAGAGTGGAATTCCACGTGTAGCGGTGAAATGCGTAGAGATGTGGAGGAACACCAGTGGCGA<br>AGGCGACTCTCTGGTCTGTAACTGACGCTGAGGAGCGAAAGCGTGGGGAGCGAACAGGATTAGATACCCTGGTAGTCCACGCCGTAAACGAT<br>GAGTGCTAAGTGTTAGGGGGTTTCCGCCCCTTAGTGCTGCAGCTAACGCATTAAGCACTCCGCCTGGGGAGTACGGTCGCAAGACTGAAACTC<br>AAAGGAATTGACGGGGGCCCGCACAAGCGGTGGAGCATGTGGTTTAATTCGAAGCAACGCGAAGAACCTTACCAGGTCTTGACATCCTCTGA<br>CAATCCTAGAAGATAGGACGTCCCCTTCGGGGGCAGAGTGACAGGTGGTGCATGGTTGTCGTCAGCTCGTGTCGTGAGATGTTGGGTTAAGTC<br>CCGCAACGAGCGCAACCCTTGATCTTAGTTGCCAGCATTCAGTTGGGCACTCTAAGGTGACTGCCGGTGACAAACCGGAGGAAGGTGGGGATG<br>ACGTCAAAT-<br>ATCATGCCCCTTATGACCTGGGCTACACACGTGCTACAATGGACAGAACAAAGGGCAGCGAAACCGCGAGGTTAAGCCAATCCCACAAATCT<br>GTTCTCAGTTCGGATCGCAGTCTGCAACTCGACTGCGTGAAGCTGGAATCGCTAGTAATCGCGGATCAGCATGCCGCGGTGAATACGTTCCCG<br>GGCCTTGTACACACCGCCCGTCACACCACGAGAGTTTGTA
S.556	PZ702558	CTCCCTGATGTTAGCGGCGGACGGGTGAGTAACACGTGGGTAACCTGCCTGTAAGACTGGGATAACTCCGGGAAACCGGGGCTAATACCGGA<br>TGGTTGTTTGAACCGCATGGTTCAGACATAAAAGGTGGCTTCGGCTACCACTTACAGATGGACCCGCGGCGCATTAGCTAGTTGGTGAGGTAA<br>CGGCTCACCAAGGCGACGATGCGTAGCCGACCTGAGAGGGTGATCGGCCACACTGGGACTGAGACACGGCCCAGACTCCTACGGGAGGCAGC<br>AGTAGGGAATCTTCCGCAATGGACGAAAGATCTGACGGAGCAACGCCGCGTGAGTGATGAAGGGGTTTTTCGGATCGTAAAAATTTGCTCTGT<br>TGTTAGGGAAGAACAAGTGCCGTTCAAATAGGGCGGCACCTTGACGGTACCTAAAACCCAGAAAGCCACGGGCTAACTACGTGCCAGCAGCC<br>GCGGTAATACGTAGGTGCGCAAGCGTTGTCCGGAATTATTGGGCGTAAAGGGCTCGCAGGCGGGTTTTTCTTAAGTCTGATGTGAAAGCCCCC

		GGGCTCAACCGGGGAGGGTCATTGGAAACTGGGGGAACTTGAGTGCAGAAGAGGAGAGTGGAATTTCCACGTGTAGCGGTGAAATGCGTAGA<br>GATGTGGAGGAACACCAGTGGCGAAGGCGACTCTCTGGTCTGTAACTGACGCTGAGGAGCGAAAGCGTGGGGAGCGAACAGGATTAGATACC<br>CTGGTAGTCCACGCCGTAAACGATGAGTGCTAAGTGTTAGGGGGTTTCCGCCCCTTAGTGCTGCAGCTAACGCATTAAGCACTCCGCCTGGGG<br>AGTACGGTCGCAAGACTGAAACTCAAAGGAATTGACGGGGGCCCGCACAAGCGGTGGAGCATGTGGTTTAATTCGAAGCAACGCGAAGAAAC<br>CTTACCAGGTCTTGACATCCTCTGACAATCCTAGAGGATAGGACGTCGCCCTTCGGGGGCAGAGTGACAGGTGGTGCATGGTTGTCTGTCAGC<br>TCGTGTCGTGAGATGTTGGGTTAAGTCC
S.593	PZ702561	AACCTGCCTGTAAGACTGGGATAACTCCGGGAAACCGGGGCTAATACCGGATGGTTGTCTGAACCGCATGGTTCAGACATAAAAGGTGGCTTC<br>GGCTACCACTTACAGATGGACCCGCGGCGCATTAGCTAGTTGGTGAGGTAACGGCTCACCAAGGCGACGATGCGTAGCCGACCTGAGAGGGT<br>GATCGGCCACACTGGGACTGAGACACGGCCCAGACTCCTACGGGAGGCAGCAGTAGGGAATCTTCCGCAATGGACGAAAGTCTGACGGAGCA<br>ACGCCGCGTGAGTGATGAAGGTTTTCGGATCGTAAAGCTCTGTTGTTAGGGAAGAACAAGTGCCGTTCAAATAGGGCGGCACCTTGACGGTAC<br>CTAACCAGAAAGCCACGGCTAACTACGTGCCAGCAGCCGCGGTAATACGTAGGTGGCAAGCGTTGTCCGGAATTATTGGGCGTAAAGGGCTC<br>GCAGGCGGTTTCTTAAGTCTGATGTGAAAGCCCCCGGCTCAACCGGGGAGGGTCATTGGAAACTGGGGAACTTGAGTGCAGAAGAGGAGAGT<br>GGAATTCCACGTGTAGCGGTGAAATGCGTAGAGATGTGGAGGAACACCAGTGGCGAAGGCGACTCTCTGGTCTGTAACTGACGCTGAGGAGC<br>GAAAGCGTGGGGAGCGAACAGGATTAGATACCCTGGTAGTCCACGCCGTAAACGATGAGTGCTAAGTGTTAGGGGGTTTCCGCCCCTTAGTGC<br>TGCAGCTAACGCATTAAGCACTCCGCCTGGGGAGTACGGTCGCAAGACTGAAACTCAAAGGAATTGACGGGGGCCCGCACAAGCGGTGGAGC<br>ATGTGGTTTAATTCGAAGCAACGCGAAGAACCTTACCAGGTCTTGACATCCTCTGACAATCCTAGAGATAGGACGTCCCCTTCGGGGGCAGAG<br>TGACAGGTGGTGCATGGTTGTCGTCAGCTCGTGTCGTGAGATGTTGGGTTAAGTCCCGCAACGAGCGCAACCCTTGATCTTAGTTGCCAGCATT<br>CAGTTGGGCACTCTAAGGTGACTGCCGGTGACAAACCGGAGGAAGGTGGGGATGACGTCAAATCATCATGCCCCTTATGACCTGGGCTACACA<br>CGTGCTACAATGGACAGAACAAAGGGCAGCGAAACCGCGAGGTTAAGCCAATCCCACAAATCTGTTCTCAGTTCGGATCGCAGTCTGCAACTC<br>GACTGCGTGAAGCTGGAATCGCTAGTAATCGCGGATCAGCATGCCGCGGTG

Table S4. ***rpoB*** **gene sequences of the selected** ***Bacillus*** **isolates in this study and their corresponding GenBank accession numbers**

Isolate code	NCBI GenBank accession number	rpoB gene sequence (5' – 3')
S.002	PZ881078	TGCTAAGCTGAGCGATGACGGTTCTTTCTTGGATGACAGCATCGTAGCGCGTTTCAGAGGGGAAAACACCGTTGTAGCCCGCAACCGCGTGGA<br>TTACATGGACGTATCTCCTAAACAGGTTGTTTCTGCTGCGACAGCATGTATTCCGTTCTTGGAAAACGATGACTCGAACCGCGCCCTCATGGGA<br>GCGAACATGCAGCGTCAGGCTGTGCCTTTGATGCAGCCGGAAGCTCCGATCGTCGGAACGGGTATGGAATATGTATCCGGTAAAGACTCCGGT<br>GCAGCCGTTATTTGTAAACACCCTGGTATCGTTGAACGGGTGGAAGCGAAAAACGTATGGGTGCGCCGCTATGAAGAAATTGACGGCCAAAA<br>AGTAAAAGGCAACCTGGATAAGTACAGCTTGCTGAAATTTGTCCGCTCCAACCAAGGTACGTGCTACAACCAGCGTCCGATCGTCAGTGTCGG<br>CGATGAAGTAGTCAAAGGAGAAATCCTTGCTGACGGACCTTCAATGGAGCTTGGTGAACTTGCTCTCGGCCGCAACGTAATGGTCGGCTTCAT<br>GACATGGGATGGTTACAACTATGAGGATGCCATCATCATGAGTGAACGCCTTGTGAAAGATGATGTATACACATCTATTCACATTGAAGAATA<br>TGAATCAGAAGCACGTGATACAAAGCTTGGGCCTGAAGAGATCACCCGCGATATTCCAAACGTAGGGGAAGACGCGCTTCGCAACCTTGATG<br>ACCGCGGAATTATCCGTATCGGTGCGGAAGTCAACGACGGAGACCTTCTCGTAGGTAAAGTAACGCCTAAAGGTGTAACTGAGCTTACGGCTG<br>AAGAACGCCTTCTGCA
S.051	PZ881079	CTTTACCCTGAAGTCGTNANCGGTAACAAGGGTGTATCGGAACGGCTTGTATGGNAACCGCCATATCGCCGCGTTGATCCTGAAACAGGGAAG<br>GTAACGGGCAGAATCGATTACTTAACTGCTGATGAAGAGGATAACTATGTTGTCGCTCAAGCAAACGCTCGTCTTGATGACGAAGGCGCCTTT<br>ATTGATGACAGCATCGTAGCTCGTTTCCGCGGGGAGAACACTGTTGTTTCCAGAAACCGTGTAGACTACATGGATGTATCGCCTAAGCAGGTT<br>GTATCTGCTGCGACAGCATGTATCCCGTTCTTAGAAAACGATGACTCCAACCGTGCCCTCATGGGAGCGAACATGCAGCGTCAGGCTGTGCCT<br>TTGATGCAGCCAGAAGCGCCATTCGTTGGAACTGGTATGGAATACGTATCAGGAAAAGACTCTGGTGCCGCTGTAATTTGTAAACACCCTGGT<br>ATCGTTGAACGCGTAGAAGCGAAAAACGTTTGGGTTCGACGTTATGAAGAAGTAGACGGTCAAAAAGTAAAAGGAAACCTGGATAAATACAG<br>CCTGCTGAAATTTGTCCGCTCTAACCAAGGTACGTGCTACAACCAGCGTCCGATCGTAAGTGTCGGCGATGAAGTGGTAAAAGGAGAAATCCT<br>TGCTGACGGTCCTTCTATGGAGCTTGGTGAACTTGCACTTGGCCGTAACGTAATGGTCGGCTTCATGACATGGGATGGTTACAACTATGAGGAT<br>GCCATCATCATGAGTGAACGCCTTGTAAAGGATGATGTTTATACATCTATCCACATTGAAGAATACGAATCAGAAGCACGTGATACGAAACTT<br>GGACCTGAAGAAATCACTCGCGATATTCCAAACGTCGGTGAAGATGCGCTTCGCAATCTTGATGACCGCGGAATCATCCGTATCGGGGCAGAA<br>GTAAAAGACGGAGATCTTCTTGTTGGTAAAGTAACGCCTAAAGGCGTAACTGAACTGACTGCTGAAGAACGCCTGCTTCACGCCATCTTTGGC

		GAGAAAGCCCGCGAGGTTCGTGATACTTCTCTCGGGTGCCTCACGGCGGCGAGGAATTATCATGACGTTAAAGTTTTCACCCTGAGAACGGGA<br>AAGAAATTCTCA
S.100	PZ881080	GCCATATCGCCGCGTTGATCCTGAAACAGGGAAGGTAACGGGCAGAATCGATTACTTAACTGCTGATGAAGAGGATAACTATGTTGTCGCTCA<br>AGCAAACGCTCGTCTTGATGACGAAGGCGCCTTTATTGATGACAGCATCGTAGCTCGTTTCCGCGGGGAGAACACTGTTGTTTCCAGAAACCG<br>TGTAGACTACATGGATGTATCGCCTAAGCAGGTTGTATCTGCTGCGACAGCATGTATCCCGTTCTTAGAAAACGATGACTCCAACCGTGCCCTC<br>ATGGGAGCGAACATGCAGCGTCAGGCTGTGCCTTTGATGCAGCCAGAAGCGCCATTCGTTGGAACTGGTATGGAATACGTATCAGGAAAAGA<br>CTCTGGTGCCGCTGTAATTTGTAAACACCCTGGTATCGTTGAACGCGTAGAAGCGAAAAACGTTTGGGTTCGACGTTATGAAGAAGTAGACGG<br>TCAAAAAGTAAAAGGAAACCTGGATAAATACAGCCTGCTGAAATTTGTCCGCTCTAACCAAGGTACGTGCTACAACCAGCGTCCGATCGTAAG<br>TGTCGGCGATGAAGTGGTAAAAGGAGAAATCCTTGCTGACGGTCCTTCTATGGAGCTTGGTGAACTTGCACTTGGCCGTAACGTAATGGTCGG<br>CTTCATGACATGGGATGGTTACAACTATGAGGATGCCATCATCATGAGTGAACGCCTTGTAAAGGATGATGTTTATACATCTATCCACATTGAA<br>GAATACGAATCAGAAGCACGTGATACGAAACTTGGACCTGAAGAAATCACTCGCGATATTCCAAACGTCGGTGAAGATGCGCTTCGCAATCTT<br>GATGACCGCGGAATCATCCGTATCGGGGCAGAAGTAAAAGACGGAGATCTTCTTGTTGGTAAAGTAACGCCTAAAGGCGTAACTGAACTGAC<br>TGCTGAAGAACGCCTGCTTCACGCCATCTTTGGCGAGAAAGCCCGCGAGGTTCGTGATACTTCTCTTCGTGTGCCTCACGGCGGCGGAGGAATT<br>ATCCATGACGTTAAAGTTTTCAACCGTGAAGAC
S.323	PZ881083	TGGTTTCATTGAGACGCCATACCGCCGCGTTGATCCTGAAACAGGAAAAGTAACGCCTAGAATCGACTACCTGACTGCTGATGAAGAGGATAA<br>CTATGTCGTAGCCCAAGCGAATGCTAAGCTGAGCGATGACGGTTCTTTCTTGGATGACAGCATCGTAGCGCGTTTCAGAGGGGAAAACACCGT<br>TGTAGCCCGCAACCGCGTGGATTACATGGACGTTTCTCCTAAACAGGTTGTTTCTGCTGCGACAGCATGTATTCCGTTCTTGGAAAACGATGAC<br>TCTAACCGTGCTCTAATGGGAGCGAACATGCAGCGTCAGGCTGTGCCTTTGATGCAGCCGGAAGCTCCGATCGTCGGAACGGGTATGGAATAC<br>GTATCCGGTAAAGACTCCGGTGCAGCCGTTATTTGTAAACACCCTGGTATCGTTGAACGGGTGGAAGCGAAAAACGTATGGGTGCGCCGCTAT<br>GAAGAAATTGACGGCCAAAAAGTAAAAGGCAACCTGGATAAGTACAGCTTGCTGAAATTTGTCCGCTCCAACCAAGGTACGTGCTACAACCA<br>GCGTCCGATCGTCAGTGTCGGCGATGAAGTAGTCAAAGGAGAAATCCTTGCTGACGGACCTTCAATGGAGCTTGGCGAACTTGCTCTCGGCCG<br>CAACGTAATGGTCGGCTTCATGACATGGGATGGTTACAACTATGAGGATGCCATCATCATGAGTGAACGCCTTGTGAAAGATGATGTATACAC<br>ATCTATTCACATTGAAGAATATGAATCAGAAGCACGTGATACAAAGCTTGGGCCTGAAGAGATCACCCGCGATATTCCAAACGTAGGGGAAG<br>ACGCGCTTCGCAATCTTGATGACCGCGGAATTATCCGTATCGGTGCGGAAGTCAACGACGGAGACCTTCTCGTAGGTAAAGTAACGCCTAAAG<br>GTGTAACTGAGCTTACGGCTGAAGAACGCC

S.374	PZ881082	TGAGACGCCATACCGCCGCGTTGATCCTGAAACAGGAAAAGTAACGCCTAGAATCGACTACCTGACTGCTGATGAAGAGGATAACTATGTCGT AGCCCAAGCGAATGCTAAGCTGAGCGATGACGGTTCTTTCTTGGATGACAGCATCGTAGCGCGTTTCAGAGGGGAAAACACCGTTGTAGCCCG CAACCGCGTGGATTACATGGACGTTTCTCCTAAACAGGTTGTTTCTGCTGCGACAGCATGTATTCCGTTCTTGGAAAACGATGACTCTAACCGT GCTCTAATGGGAGCGAACATGCAGCGTCAGGCTGTGCCTTTGATGCAGCCGGAAGCTCCGATCGTCGGAACGGGTATGGAATACGTATCCGGT AAAGACTCCGGTGCAGCCGTTATTTGTAAACACCCTGGTATCGTTGAACGGGTGGAAGCGAAAAACGTATGGGTGCGCCGCTATGAAGAAATT GACGGCCAAAAAGTAAAAGGCAACCTGGATAAGTACAGCTTGCTGAAATTTGTCCGCTCCAACCAAGGTACGTGCTACAACCAGCGTCCGATC GTCAGTGTCGGCGATGAAGTAGTCAAAGGAGAAATCCTTGCTGACGGACCTTCAATGGAGCTTGGCGAACTTGCTCTCGGCCGCAACGTAATG GTCGGCTTCATGACATGGGATGGTTACAACTATGAGGATGCCATCATCATGAGTGAACGCCTTGTGAAAGATGATGTATACACATCTATTCAC ATTGAAGAATATGAATCAGAAGCACGTGATACAAAGCTTGGGCCTGAAGAGATCACCCGCGATATTCCAAACGTAGGGGAAGACGCGCTTCG CAATCTTGATGACCGCGGAATTATCCGTATCGGTGCGGAAGTCAACGACGGAGACCTTCTCGTAGGTAAAGTAACGCCTAAAGGTGTAACTGA GCTTACGGCTGAANAACGCCTTCTGCATGCGATCTTTGGAGAAAAAGCGCGTGAAGTCCGTGATACTTCTCTCCGTGTGCCTCACGGCGGCGG CGGAATTATCCACGACGTAAAAGTCTTCAACCGTGAAG
S.409	PZ881087	GATCAACTCACTTTCATCTTATGCAAAAGTAAACCGTTGTTGGCGTTTATTGAAACGCCATATCGCCGCGTTGACCCTGAAACAGGGAAGGTA ACGGGCAGAATCGATTACTTAACTGCTGATGAAGAGGATAACTATGTTGTCGCTCAAGCGAATGCTCGTCTTGATGACGAAGGCGCCTTTATT GATGACAGCATCGTAGCTCGTTTCCGCGGGGAGAACACTGTTGTTTCCAGAAATCGTGTAGACTACATGGATGTATCGCCTAAGCAGGTTGTA TCTGCTGCGACAGCATGTATCCCGTTCTTAGAAAACGATGACTCCAACCGTGCCCTCATGGGAGCGAACATGCAGCGTCAGGCTGTGCCTTTG ATGCAGCCGGAAGCGCCATTTGTTGGAACTGGTATGGAATACGTATCAGGAAAAGACTCTGGTGCCGCTGTTATTTGTAAACACCCTGGTATC GTTGAACGCGTAGAAGCGAAAAACGTTTGGGTTCGCCGTTATGAAGAAGTAGACGGTCAAAAAGTAAAAGGAAACCTGGATAAATACAGCCT GCTGAAATTTGTCCGCTCTAACCAAGGTACGTGCTACAACCAGCGTCCGATCGTAAGTGTCGGCGATGAAGTGGTAAAAGGAGAAATCCTTGC TGACGGTCCTTCTATGGAGCTTGGTGAACTTGCACTTGGCCGTAACGTAATGGTCGGCTTCATGACATGGGATGGCTACAACTATGAGGATGCC ATCATCATGAGTGAACGCCTAGTGAAGGATGATGTTTATACATCTATCCACATTGAAGAATACGAATCAGAAGCACGTGATACGAAACTTGGA CCTGAAGAAATCACTCGCGATATTCCAAACGTCGGTGAAGATGCGCTTCGCAATCTTGATGACCGCGGAATCATCCGTATTGGGGCAGAAGTA AAAGACGGAGATCTTCTTGTTGGTAAAGTAACGCCTAAAGGCGTAACTGAACTGACTGCAGAAGAACGCCTTCTTCATGCCATCTTTGGCGAG AAAGCCCGCGAGGTTCGTGATACTTCTCTTCGTGTGCCTCATGGCGGCGGCGGAA

S.441	PZ881085	TTTGATGCAGCCAGAAGCGCCATTCGTTGGAACTGGTATGGAATACGTATCAGGAAAAGACTCTGGTGCCGCTGTAATTTGTAAACACCCTGG<br>TATCGTTGAACGCGTAGAAGCGAAAAACGTTTGGGTTCGACGTTATGAAGAAGTAGACGGTCAAAAAGTAAAAGGAAACCTGGATAAATACA<br>GCCTGCTGAAATTTGTCCGCTCTAACCAAGGTACGTGCTACAACCAGCGTCCGATCGTAAGTGTCGGCGATGAAGTGGTAAAAGGAGAAATCC<br>TTGCTGACGGTCCTTCTATGGAGCTTGGTGAACTTGCACTTGGCCGTAACGTAATGGTCGGCTTCATGACATGGGATGGTTACAACTATGAGGA<br>TGCCATCATCATGAGTGAACGCCTTGTAAAGGATGATGTTTATACATCTATCCACATTGAAGAATACGAATCAGAAGCACGTGATACGAAACT<br>TGGACCTGAAGAAATCACTCGCGATATTCCAAACGTCGGTGAAGATGCGCTTCGCAATCTTGATGACCGCGGAATCATCCGTATCGGGGCAGA<br>AGTAAAAGACGGAGATCTTCTTGTTGGTAAAGTAACGCCTAAAGGCGTAACTGAACTGACTGCTGAAGAACGCCTGCTTCACGCCATCTTTGG<br>CGAGAAAGCCCGCGAGGTTCGTGATACTTCTCTTCGTGTGCCTCACGGCGGCGGAGGAATTATCCATGACGTTAAAGTTTTCAACCGTGAAGA
S.467	PZ881081	CCGCTTTGGGTTTCATTGAGACGCCATACCGCCGCGTTGATCCTGAAACAGGAAAAGTAACGCCTAGAATCGACTACCTGACTGCTGATGAAG<br>AGGATAACTATGTCGTAGCCCAAGCGAATGCTAAGCTGAGCGATGACGGTTCTTTCTTGGATGACAGCATCGTAGCGCGTTTCAGAGGGGAAA<br>ACACCGTTGTAGCCCGCAACCGCGTGGATTACATGGACGTATCTCCTAAACAGGTTGTTTCTGCTGCGACAGCATGTATTCCGTTCTTGGAAAA<br>CGATGACTCGAACCGCGCCCTCATGGGAGCGAACATGCAGCGTCAGGCTGTGCCTTTGATGCAGCCGGAAGCTCCGATCGTCGGAACGGGTAT<br>GGAATATGTATCCGGTAAAGACTCCGGTGCAGCCGTTATTTGTAAACACCCTGGTATCGTTGAACGGGTGGAAGCGAAAAACGTATGGGTGCG<br>CCGCTATGAAGAAATTGACGGCCAAAAAGTAAAAGGCAACCTGGATAAGTACAGCTTGCTGAAATTTGTCCGCTCCAACCAAGGTACGTGCTA<br>CAACCAGCGTCCGATCGTCAGTGTCGGCGATGAAGTAGTCAAAGGAGAAATCCTTGCTGACGGACCTTCAATGGAGCTTGGTGAACTTGCTCT<br>CGGCCGCAACGTAATGGTCGGCTTCATGACATGGGATGGTTACAACTATGAGGATGCCATCATCATGAGTGAACGCCTTGTGAAAGATGATGT<br>ATACACATCTATTCACATTGAAGAATATGAATCAGAAGCACGTGATACAAAGCTTGGGCCTGAAGAGATCACCCGCGATATTCCAAACGTAGG<br>GGAAGACGCGCTTCGCAATCTTGATGACCGCGGAATTATCCGTATCGGTGCGGAAGTCAACGACGGAGACCTTCTCGTAGGTAAAGTAACGCC<br>TAAAGGTGTAACTGAGCTTACGGCTGAAGAACGCCTTCTGCATGCGATCTTTGGAGAAAAAGCGCGTGAAGTCCGTGATACTTCTCTCCGTGT<br>GCCTCACGGCGGCGGCGGAATTATCCACGACGTAAAAGTCTTCAACCGTGAAGACGGCGACGAACTTCCTCCGGG
S.512	PZ881086	CCGTTTTGGCTTTATTGAAACGCCATATCGCCGCGTTGACCCTGAAACAGGGAAGGTAACGGGCAGAATCGATTACTTAACTGCTGATGAAGA<br>GGATAACTATGTTGTCGCTCAAGCGAATGCTCGTCTTGATGACGAAGGCGCCTTTATTGATGACAGCATCGTAGCTCGTTTCCGCGGGGAGAA<br>CACCGTTGTTTCCAGAAATCGTGTAGACTACATGGATGTATCGCCTAAGCAGGTTGTATCTGCTGCGACAGCATGTATCCCGTTCTTAGAAAAC<br>GATGACTCCAACCGTGCCCTCATGGGAGCGAACATGCAGCGTCAGGCTGTGCCTTTGATGCAGCCGGAAGCGCCATTTGTTGGAACTGGTATG<br>GAATACGTATCAGGAAAAGACTCTGGTGCCGCTGTTATTTGTAAACACCCTGGTATCGTTGAACGCGTAGAAGCGAAAAACGTTTGGGTTCGC<br>CGTTATGAAGAAGTAGACGGTCAAAAAGTAAAAGGAAACCTGGATAAATACAGCCTGCTGAAATTTGTCCGCTCTAACCAAGGTACGTGCTA

		CAACCAGCGTCCGATCGTAAGTGTCGGCGATGAAGTGGTAAAAGGAGAAATCCTTGCTGACGGTCCTTCTATGGAGCTTGGTGAACTTGCACT TGGCCGTAACGTAATGGTCGGCTTCATGACATGGGATGGCTACAACTATGAGGATGCCATCATCATGAGTGAACGCCTAGTGAAGGATGATGT TTATACATCTATCCACATTGAAGAATACGAATCAGAAGCACGTGATACGAAACTTGGACCTGAAGAAATCACTCGCGATATTCCAAACGTCGG TGAAGATGCGCTTCGCAATCTTGATGACCGCGGAATCATCCGTATTGGGGCAGAAGTAAAAGACGGAGATCTTCTTGTTGGTAAAGTAACGCC TAAAGGCGTAACTGAACTGACTGCAGAAGAACGCCTTCTTCACGCCATCTTTGGCGAGAAAGCCCGCGAGGTTCGTGATACTTCTCTTCGTGTG CCTCATGGCGGCGGCGGAATTATCCATGACGTTAAAGTCTTCAACCGTGAAGACGGAGACGAACTTCCTCCAGGTGT
S.556	PZ881084	CAGGAAAAGTAACGCCTAGAATCGACTACCTGACTGCTGATGAAGAGGATAACTATGTCGTAGCCCAAGCGAATGCTAAGCTGAGCGATGAC GGTTCTTTCTTGGATGACAGCATCGTAGCGCGTTTCAGAGGGGAAAACACCGTTGTAGCCCGCAACCGCGTGGATTACATGGACGTATCTCCT AAACAGGTTGTATCTGCTGCGACAGCATGTATTCCGTTCTTGGAAAACGATGACTCGAACCGCGCCCTCATGGGAGCGAACATGCAGCGTCAG GCTGTGCCTTTGATGCAGCCGGAAGCTCCGATCGTCGGAACGGGTATGGAATACGTATCCGGTAAAGACTCCGGTGCAGCCGTTATTTGTAAA CACCCTGGTATCGTAGAACGGGTGGAAGCGAAAAACGTATGGGTGCGCCGCTATGAAGAAATTGACGGCCAAAAAGTAAAAGGCAACCTGGA TAAGTACAGCTTGCTGAAATTTGTCCGCTCCAACCAAGGTACGTGCTACAACCAGCGTCCGATCGTCAGTGTCGGCGATGAAGTAGTCAAAGG AGAAATCCTTGCTGACGGACCTTCAATGGAGCTTGGTGAACTTGCTCTCGGCCGCAACGTAATGGTCGGCTTCATGACATGGGACGGTTACAA CTATGAGGATGCCATCATCATGAGTGAACGCCTTGTGAAAGATGATGTATACACATCTATTCACATTGAAGAATATGAATCAGAAGCACGTGA TACAAAGCTTGGACCGGAAGAGATCACCCGCGATATTCCAAACGTAGGGGAAGACGCGCTTCGCAACCTTGATGACCGTGGAATTATCCGTAT CGGTGCGGAAGTCAACGACGGAGACCTTCTCGTAGGTAAAGTAACGCCTAAAGGTGTAACTGAGCTTACGGCTGAAGAACGCCTTCTTCATGC GATCTTTGGAGAAAAAGCGCGTGAGGTCCGTGATACTTCTCTCCGTGTGCCTCACGGCGGCGGCGGAATTATCCACGACGTAAAAGTCTTCAA CCGTGAAGACG
S.593	PZ881088	GTGGATTACATGGACGTATCTCCTAAACAGGTTGTTTCTGCTGCGACAGCATGTATTCCGTTCTTGGAAAACGATGACTCGAACCGCGCCCTCA TGGGAGCGAACATGCAGCGTCAGGCTGTGCCTTTGATGCAGCCGGAAGCTCCGATCGTCGGAACGGGTATGGAATACGTATCCGGTAAAGACT CCGGTGCAGCCGTTATTTGTAAACACCCTGGTATCGTAGAACGGGTGGAAGCGAAAAACGTATGGGTGCGCCGCTATGAAGAAATTGACGGC CAAAAAGTAAAAGGCAACCTGGATAAGTACAGCTTGCTGAAATTTGTCCGCTCCAACCAAGGTACGTGCTACAACCAGCGTCCGATTGTAAGT GTCGGCGATGAAGTAGTCAAAGGAGAAATCCTTGCTGACGGACCTTCAATGGAGCTTGGTGAACTTGCTCTCGGCCGCAACGTAATGGTCGGC TTCATGACATGGGACGGTTACAACTATGAGGATGCCATCATCATGAGTGAACGCCTTGTGAAAGATGATGTATACACATCTATTCACATTGAA GAATATGAATCAGAAGCACGTGATACAAAGCTTGGACCGGAAGAGATCACCCGCGATATTCCAAACGTAGGGGAAGACGCGCTTCGCAACCT

		TGATGACCGCGGAATTATCCGTATCGGTGCGGAAGTCAACGACGGAGACCTTCTCGTAGGTAAAGTAACGCCTAAAGGTGTAACTGAGCTTAC GGCTGAAGAACGCCTTCTTCATGCGA

Table S5. LC–MS/MS characterization and tentative identification of compounds detected (in fraction F4) in the ethyl acetate extract of *Bacillus velezensis* S.002.

No.	Retention time (min)	Putatively identified compound	Ion	Precursor ion (m/z)	Fragment ions (m/z)	Molecular formula (mass error, ppm)	Cosine	Molecular structure
1	5,88	cyclo(L-Phe-D-Pro)	M+H	245.128	70 (100), 98, 120, 154,	N/A (1,49396)	0.965989	
2	2,17	Cyclo(-His-Pro)	M+H	235.119	70, 82, 93, 110 (100), 235	$C_{11}H_{14}N_4O_2$ (0,51919)	0.96447	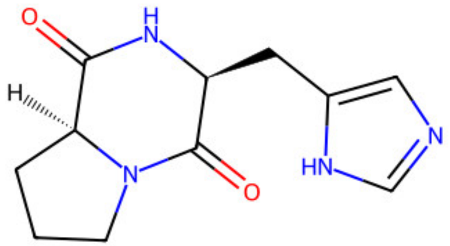

3	8,4	Norharmane	[M+H]+	169.077	115, 169 (100)	$C_{11}H_8N_2$ (5,95634)	0.953111	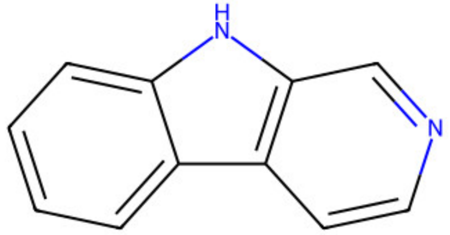
4	16,66	Thr-C14:0 (N-tetradecanoylthreonine)	M+H	330.264	56, 74 (100), 102, 120	$C_{18}H_{35}NO_4$ (0,64683)	0.950665	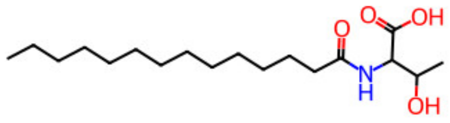

5	17,83	Ser-C16:0 (N-hexadecanoylserine)	M+H	344.28	60 (100), 88, 106	$C_{19}H_{37}NO_4$ (2,03876)	0.94919	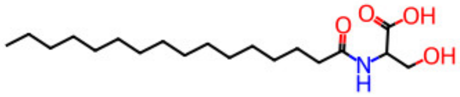
6	18,18	Thr-C16:0 (N-hexadecanoylthreonine)	M+H	358.295	56, 74 (100), 102, 120	$C_{20}H_{39}NO_4$ (0,08517)	0.938532	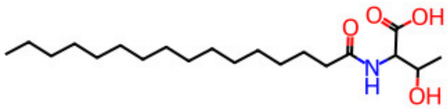

7	5,64	Cyclo(proline-leucine)	M+H	211.144	70 (100), 86, 98, 211	$C_{11}H_{18}N_2O_2$ (0,79494)	0.919528	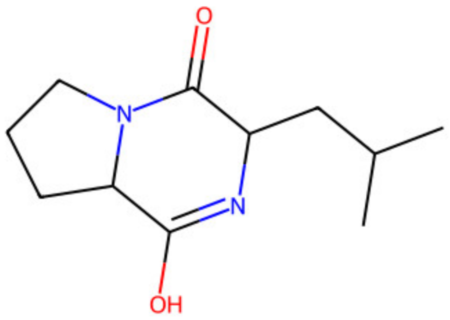
8	2,82	Quinoline-4-carboxylic acid	[M+H]+	174.055	103, 130, 146, 174 (100)	$C_{10}H_7NO_2$ (0)	0.899307	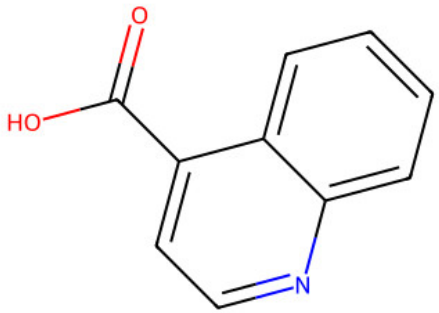

9	15,11	Leu-C15:1 (N-pentadecenoylleucine)	M+H	354.3	55, 69, 86 (100), 132	$C_{21}H_{39}NO_3$ (0,60294)	0.894121	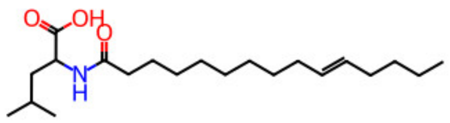
10	16,98	Ser-C15:0 (N-pentadecanoylserine)	M+H	330.264	60 (100), 88, 106	$C_{18}H_{35}NO_4$ (0,92404)	0.89149	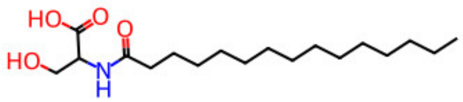

11	14,71	Ser-C16:1 (N-hexadecenoylserine)	M+H	342.264	60 (100), 88, 106	$C_{19}H_{35}NO_4$ (0,89164)	0.864681	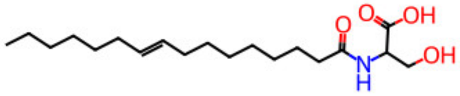
12	22,85	Surfactin C	M+H	1036.69	86 (100)	$C_{53}H_{93}N_7O_{13}$ (0,11775)	0.859055	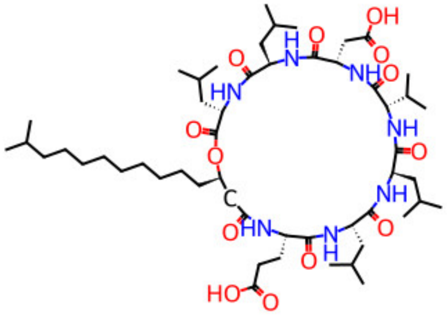

13	16,84	Ser-C18:1 (N-octadecenoylserine)	M+H	370.295	60 (100), 88, 106	$C_{21}H_{39}NO_4$ (0,65931)	0.841027	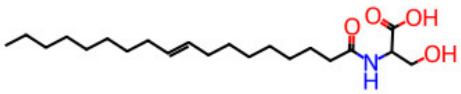
14	21,03	Surfactin B	M+H	1022.67	86 (100), 199	$C_{52}H_{91}N_7O_{13}$ (4,11807)	0.818123	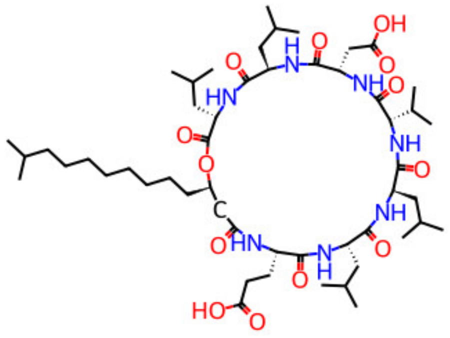

15	15,2	cyclo(L-Leu-L-4-Hyp)	M+H	227.139	53, 68, 86 (100), 114, 227	N/A (0,33589)	0.815701	
16	14,07	Thr-C16:1 (N-hexadecenoylthreonine)	M+H	356.28	56, 74 (100), 102, 120	$C_{20}H_{37}NO_4$ (2,05575)	0.798121	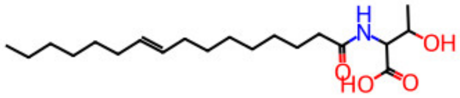

17	13,99	3-hydroxyhexadecanoyl threonine [M+H]+	[M+H]+	374.29	56, 74 (100), 102, 120	$C_{20}H_{39}NO_5$ (0,16307)	0.797112	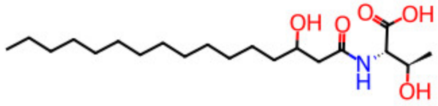
18	16,23	Serine-C18:2 (N-octadecadienoylserine)	M+H	368.279	60 (100), 81, 95, 106	$C_{21}H_{37}NO_4$ (1,65731)	0.790736	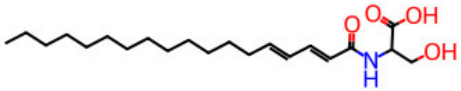

19	7,14	Tryptophan	M+H	205.097	55, 65, 72, 91, 103, 118 (100), 132, 146, 169, 188, 205	$C_{11}H_{12}N_2O_2$ (1,41356)	0.78987	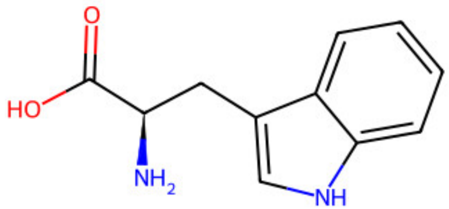
20	13,78	3-hydroxyhexadecanoyl serine [M+H]+	[M+H]+	360.274	60 (100), 88, 106	$C_{19}H_{37}NO_5$ (0,84707)	0.789767	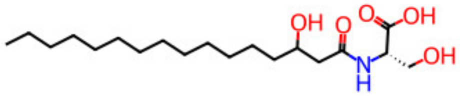

21	5,61	cyclo(Phe-Leu)	M+H	261.159	70, 86, 103, 120 (100), 188	$C_{15}H_{20}N_2O_2$ (2,5708)	0.774962	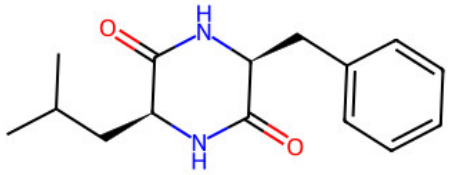
22	12,99	Bacillomycin LC2	[M+H]+	1035.54	70, 86, 103, 120 (100), 188	$C_{47}H_{74}N_{10}O_{16}$ (5,18676)	0.751877	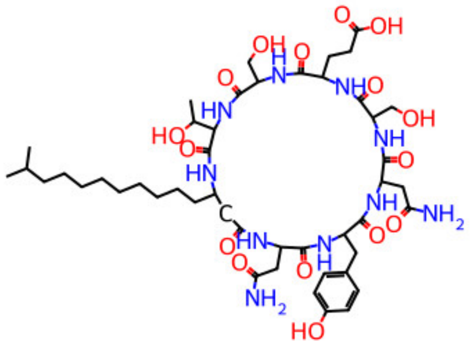

23	5,32	PyroGlu-Pro	M+H	227.103	91, 136 (100), 198, 299,	$C_{10}H_{14}N_2O_4$ (1,74691)	0.708042	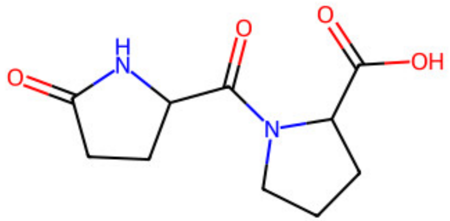
24	19,01	Surfactin A	M+H	1008.66	86 (100), 199	$C_{51}H_{89}N_7O_{13}$ (1,02869)	0.704301	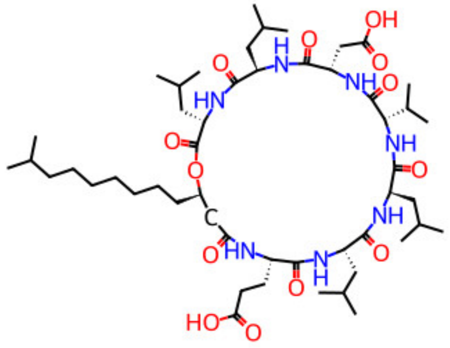

Note: Identification confidence corresponds to MSI Level 2 (putatively annotated compounds based on accurate mass error < 5 ppm and GNPS MS/MS spectral library matching with manual verification of fragmentation patterns) and MSI Level 3 (putatively characterized compound classes) when positional isomers or stereoisomers cannot be unambiguously discriminated.
